# Efficacy and safety of preoperative IOP reduction using a preservative-free fixed combination of dorzolamide/timolol eye drops versus oral acetazolamide and dexamethasone eye drops and assessment of the clinical outcome of trabeculectomy in glaucoma

**DOI:** 10.1371/journal.pone.0171636

**Published:** 2017-02-15

**Authors:** Katrin Lorenz, Joanna Wasielica-Poslednik, Katharina Bell, Giulia Renieri, Alexander Keicher, Christian Ruckes, Norbert Pfeiffer, Hagen Thieme

**Affiliations:** 1 Department of Ophthalmology, University Medical Center, Johannes Gutenberg-University Mainz, Mainz, Germany; 2 University Eye Clinic, Otto-von-Guericke University Magdeburg, Magdeburg, Germany; 3 Augenärzte im Basteicenter, Ulm, Germany; 4 Interdisciplinary Center Clinical Trials Mainz, University Medical Center, Johannes Gutenberg-University Mainz, Mainz, Germany; Cardiff University, UNITED KINGDOM

## Abstract

**Introduction:**

To demonstrate that preoperative treatment for 28 days with topical dorzolamide/timolol is non-inferior (Δ = 4 mm Hg) to oral acetazolamide and topical dexamethasone (standard therapy) in terms of intraocular pressure (IOP) reduction 3 and 6 months after trabeculectomy in glaucoma patients.

**Materials and methods:**

Sixty-two eyes undergoing trabeculectomy with mitomycin C were included in this monocentric prospective randomized controlled study. IOP change between baseline and 3 months post-op was defined as the primary efficacy variable. Secondary efficacy variables included the number of 5-fluorouracil (5-FU) injections, needlings, suture lyses, preoperative IOP change, hypertension rate and change of conjunctival redness 3 and 6 months post-op. Safety was assessed based on the documentation of adverse events.

**Results:**

Preoperative treatment with topical dorzolamide/timolol was non-inferior to oral acetazolamide and topical dexamethasone in terms of IOP reduction 3 months after trabeculectomy (adjusted means -8.12 mmHg versus -8.30 mmHg; Difference: 0.18; 95% CI -1.91 to 2.26, p = 0.8662). Similar results were found 6 months after trabeculectomy (-9.13 mmHg versus -9.06 mmHg; p = 0.9401). Comparable results were also shown for both groups concerning the classification of the filtering bleb, corneal staining, and numbers of treatments with 5-FU, needlings and suture lyses. More patients reported AEs in the acetazolamide/dexamethasone group than in the dorzolamide/timolol group.

**Discussion:**

Preoperative, preservative-free, fixed-dose dorzolamide/timolol seems to be equally effective as preoperative acetazolamide and dexamethasone and has a favourable safety profile.

## Introduction

The most common cause of post-trabeculectomy filtration failure is postoperative scarring [1]. Long-term preoperative treatment with topical antiglaucoma agents containing preservatives, usually benzalkonium chloride, leads to histologic changes of the conjunctiva, with increased numbers and activity of lymphocytes, monocytes, fibroblasts, and conjunctival goblet cells. These changes may favour the development of filtration bleb scarring [2–4] and increase the rate of post-trabeculectomy filtration failure [5–7]. Given these considerations, Broadway et al proposed discontinuing antiglaucoma drugs and treating patients preoperatively with topical steroids. Many surgeons therefore prepare their patients for planned trabeculectomy by stopping topical antiglaucoma drugs, controlling intraocular pressure (IOP) with oral acetazolamide, and treating the eye with topical steroids [8]. Breusegem et al [9] showed the usefulness of this approach in a randomized, controlled, three-arm study. This preoperative treatment led to fewer filtering bleb revisions postoperatively. In addition, fewer IOP-lowering medications were necessary. Preoperative treatment still differs significantly between centres. The results published to date have often been based on retrospective, nonrandomized, nonmasked investigations and have not studied the latest generation of topical antiglaucoma drugs [10]. Therefore, a retrospective study with 26 glaucoma patients undergoing trabeculectomy [11] has been conducted.

The currently used preoperative preparation in Mainz and other centres [12] has several potential disadvantages [13–15] We therefore sought an alternative preoperative treatment that would be better tolerated by patients in terms of safety and quality of life, that would require less visits to the ophthalmologist and general practitioner and would therefore be cheaper, and is that would be equally effective in reducing IOP three and six months after trabeculectomy. No treatment guidelines yet exist for the pretreatment prior to trabeculectomy. Following recommendations by Klink et al [12], we decided to use preservative-free ß-blockers and/ or carbonic anhydrase inhibitors as a comparator.

This study investigated the efficacy and safety of preoperative IOP reduction using two different IOP lowering pharmaceutical interventions prior to trabeculectomy: topical preservative-free dorzolamide/timolol eye drops (COSOPT-S^®^) (previously MSD SHARP & DOHME GmbH, Germany; now Santen OY, Finland) compared to oral acetazolamide plus topical dexamethasone. This trial also sought to determine whether the preoperative treatment affects postoperative outcome (IOP and scarring), the safety and tolerability of the two interventions, whether patients prefer one or the other of the tested therapies and health-related economic aspects.

## Patients and methods

**Fig 1:**
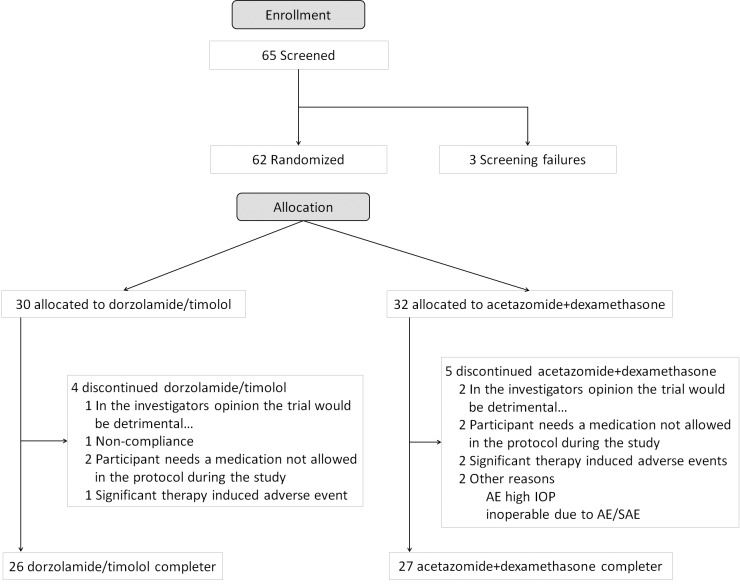
Consort diagram.

This prospective, randomized, monocentic, controlled study was approved by the responsible ethics committee (Landesärztekammer Rheinland-Pfalz, Mainz, Germany) and the competent authority (BfArM, Germany). The study was registered at clinicaltrials.gov before the first patient was included (ClinicalTrials.gov Identifier: NCT01228149). Signed and dated informed consent of the subject was available before the start of any specific trial procedures. Sixty-two eyes of 62 patients undergoing standardized trabeculectomy with intraoperative application of mitomycin C (100 μl, 0.2 mg/ml for 5 minutes) by 3 experienced surgeons in the Department of Ophthalmology, University Medical Center, Johannes Gutenberg-University Mainz, Germany were included (first patient in: 23 SEP 2010; last patient in: 02 OCT 2013; last patient out: 23 APR 2014) (Fig 1). The study treatment was administered over 4 weeks before trabeculectomy. Patients were followed up for 24 weeks after surgery. Before the first patient was enrolled in the trial, ethics approval and competent authority approval was sought in accordance with local legal requirements. The in- and exclusion criteria are summarized in Table 1.

**Table 1:** In- and exclusion criteria.

	**Inclusion Criteria**
1	Male or female patients aged 18 years or older
2	Caucasian
3	A clinical diagnosis of open-angle glaucoma, pseudoexfoliative or pigment dispersion glaucoma, or ocular hypertension in one or both eyes
4	Planned trabeculectomy with mitomycin C (MMC)
5	Previous treatment with antiglaucoma agents containing preservatives for at least one month
6	Best corrected visual acuity of 20/800 or better in the study eye
7	Ability of subject to understand the character and individual consequences of the clinical trial
8	Signed and dated informed consent of the subject was available before the start of any specific trial procedures
9	Women with childbearing potential had to practice medically accepted contraception during the trial, and a negative pregnancy test (serum or urine) had to exist before the trial.
	**Exclusion Criteria**
1	Secondary glaucoma except pseudoexfoliative glaucoma and pigmentary glaucoma
2	Current ocular infection; i.e., conjunctivitis or keratitis
3	Any abnormality preventing reliable applanation tonometry
4	Intraocular surgery or laser treatment within the past three months
5	History of surgery involving the conjunctiva
6	History of cataract surgery using the sclerocorneal approach
7	Allergy to sulphonamides
8	Reactive airway disease, including bronchial asthma or a history of bronchial asthma, or severe chronic obstructive pulmonary disease
9	Sinus bradycardia, second or third degree atrioventricular block, overt cardiac failure, or cardiogenic shock
10	Severe renal dysfunction (CrCl < 30 ml/min) or hyperchloraemic acidosis
11	Depressed blood levels of sodium and/or potassium
12	Marked kidney and liver disease or dysfunction, gout, suprarenal gland failure, hypercalcinuria or nephrocalcinosis
13	History of hypersensitivity to the investigated medicinal products or to any drug with similar chemical structure or to any excipient present in the pharmaceutical formulation of the investigated medicinal products
14	Participation in other clinical trials during the present clinical trial or within the last four weeks
15	Medical or psychological conditions that would not permit completion of the trial or the signing of informed consent
16	Pregnancy and lactation

The following study treatments were given:

Test therapy:

Twenty-eight days preoperatively (± 3 days): Discontinuation of all topical antiglaucomatous drugs and IOP regulation with dorzolamide 20 mg/ml and timolol 5 mg/ml, COSOPT-S^®^ eye drops (previously MSD SHARP & DOHME, Munich, Germany; now Santen OY, Tampere, Finland) b.i.d. (7:00 am, 7:00 pm; +/- 1 h).

Reference therapy:

Preoperative standard management of the Department of Ophthalmology, University Medical Center, Johannes Gutenberg-University Mainz, Germany:

Twenty-eight days preoperatively (± 3 days): Discontinuation of all topical antiglaucomatous drugs and IOP regulation with oral acetazolamide (Diamox^®^ 250 mg tablets; Mercury Pharmaceuticals Ltd., London, UK) 250 mg—1500 mg per day, two or three doses, (dosage depending on IOP and disease severity at the discretion of the investigator)); potassium monitoring and potassium replacement if required.

Seven days preoperatively (±1 day): preservative-free dexamethasone 1 mg/ml; (DexaEDO^®^ eye drops, Dr. Gerhard Mann Chem.-pharm. Fabrik GmbH, Berlin, Germany) five times daily in the studied eye. Patients were randomized to dorzolamide/timolol treatment or standard therapy (1:1) using permuted blocks without stratification factors.

The following concomitant treatments were not permitted during the trial: The use of any systemic medication that would affect IOP with a stable dosing regimen of less than one month before the screening visit (i.e., steroids) and the use of any additional topical treatment that affects IOP. Patients were instructed to bring all trial medications to the trial site at every visit. Compliance was assessed by reviewing the patient’s follow-up booklet, counting the drug bottles and measuring the bottles' weight.

Trial schedule: see Table 2.

**Table 2:** Trial schedule.

Visit and Action	Visit 1 (Screening & Randomization)	Visit 2	Surgery	Visit 3	Visit 4	Visit 5	Visit 6
Trial day	-16 weeks to -28 days	Day -1	Day 0	Day 7±3	Day 28±7	Week 12±7days	Week 24±14 days
Demographics (sex, age)	X						
Patient information and informed consent	X						
Previous and concomitant diseases	X						
Previous and concomitant treatments	X						
Inclusion/exclusion criteria	X						
Pregnancy Test (if applicable)	X						
Laboratory tests (if applicable)	X						
Trabeculectomy			X				
Best-corrected visual acuity (ETDRS charts)	X	X		X	X	X	X
Non-contact Pachymetry		X		X		X	
IOP	X	X		X	X	X	X
Optical Coherence Tomography		X		X	X	X	X
Axial length (IOL Master)		X			X		
Anterior chamber depth (AC or IOL Master)		X		X			
Photography (conjunctiva)	X	X				X	X
Slit lamp examination	X	X		X	X	X	X
Randomization	X						
Filtration bleb classification (Grehn)				X	X	X	X
Grading of conjunctiva (ORA redness scale)	X	X					
Questionnaires (Patients’ satisfaction, NEI VFQ-25)	X	X				X	
Previous 5-FU injections and suture lyses						X	
Previous needlings						X	
Changes in medical health or concomitant medication		X		X	X	X	X
Adverse events		X		X	X	X	X
End of trial (final visit)							X

IOP was determined once at each visit using Goldmann applanation tonometry at the same time (±1 h) by an investigator without a blinded observer. IOP change between baseline and three months post-OP was defined as the primary efficacy variable.

Secondary efficacy variables were as follows:

- Number of 5-FU injections and suture lyses up to Visits 5 and 6
- Number of needlings and reoperations up to Visits 5 and 6
- IOP change between Visits 1 and 2
- Comparison between both groups of ocular hypotension rate (IOP 0–5 mm Hg) and filtration bleb classification in both groups at every postoperative visit
- Conjunctival status change between Visits 1 and 2 in both groups (assessed using the ORA redness scale and the filtration bleb classification)

Safety was assessed based on the documentation of adverse events (AEs) and serious adverse events (SAEs).

For statistical analysis, SAS version 9.2 was used. Parameters were described using the following descriptive statistics: Quantitative variables: N with data, mean, standard deviation, median, minimum and maximum. Categorical variables: frequencies and percentages. Outliers were not investigated, and missing values were not substituted. IOP change in the studied eye between visit 1 and three months post-OP (visit 5) was defined as the primary efficacy variable. Variables were not transformed before fitting the statistical model. A non-inferiority test was carried out to show that treatment with dorzolamide/timolol was not inferior to the standard preoperative treatment. An ANCOVA model was implied including the IOP baseline value as a covariate. Δ = 4 mm Hg was pre-specified as the non-inferiority margin. The 95% confidence interval for treatment differences was calculated. If the lower 95% confidence limit was located above the non-inferiority margin, Δ non-inferiority was concluded. Additionally, IOP values by visit and treatment group were displayed using box-whisker plots. The primary analysis was conducted for the per protocol (PP) population, which was considered as the conservative analysis population in this specific non-inferiority setting and comprised all patients who were treated for more than 8 days and who did not take any medication that interfered with the study medication during this time frame; the analysis was repeated for the intention to treat (ITT) population. Post-hoc, we calculated additional p-values for possible other non-inferiority bounds: 3 mmHg, 2.5 mmHg, and 2.0 mmHg.

Secondary analyses comprised ANCOVAs or Wilcoxon tests for continuous variables. For the NEI VFQ25, models accounting for repeated measurements were used. Rates were analysed using chi-square tests. All secondary analyses were exploratory and were interpreted based on a two-sided level of significance of α = 0.05.

The sample size was chosen using SAS V9.2 with a non-inferiority margin of 4 mmHg, a common standard deviation of 5 mmHg, a power of 80% and a one-sided significance level of α = 0.025. For planning purposes, a t-test was used, which resulted in a total sample size of 52 evaluable patients. Considering 10% of the patients as not evaluable per protocol, 60 patients were planned for inclusion in the study.

## Results

In total, 62 patients were randomized; 30 were assigned to the dorzolamide/timolol treatment and 32 were assigned to the acetazolamide/dexamethasone treatment. All of these patients were included in the ITT population. The PP population comprised 58 patients with 27 patients in the dorzolamide/timolol arm and 31 patients in the acetazolamide/dexamethasone arm and constituted patients without any major protocol violation. Fifty-three patients completed the study according to the protocol (see S1 File. Clinical Trial Protocol). Reasons for discontinuation were as follows: the use of forbidden medication during the trial and adverse events, such as high IOP and non-compliance. Patient data is summarized in Table 3.

**Table 3:** Patient data. a) General patient information, b) Distribution of glaucoma diagnosis in population, c) Previous use of IOP-lowering eye treatments.

***a)***			
	**dorzolamide/timolol arm**	**acetazolamide/dexamethasone arm**	**Total**
Patient number (ITT population)	N = 30	N = 32	N = 62
Patient number (PP popoulation)	N = 27	N = 31	N = 58
Study completion according to protocol	N = 26	N = 27	N = 53
Mean age [years]	65.8 (±8.82)	64.2 (±10.51)	
Sex	female 40.0%; male 60.0%	female 59.38%; male 40.63%	female N = 31; male N = 31
***b)***			
POAG		N = 49 (79.03%)	
glaucoma other than POAG:		N = 13 (20.97%)	
• Pseudoexfoliative glaucoma		N = 6 (9.68%)	
• Pigmentary glaucoma		N = 3 (4.84%)	
• Normal-tension glaucoma		N = 3 (4.84%)	
• Primary angle-closure glaucoma		N = 1 (1.61%)	
***c)***			
**Medication**	**Number of patients**		**% of ITT**
ß-blocking agents	49		79.03
carbonic anhydrase inhibitors	26		41.94
prostaglandin analogues	44		70.97
adrenergic agonists	41		66.13

All patients reported the previous use of IOP-lowering eye treatments in the studied eye (Table 3c). We observed no relevant differences between the treatment groups. In the dorzolamide/timolol treatment group, the IOP in the studied eye changed from 17.4 mmHg to 10.5 mmHg after 3 months; in the acetazolamide/dexamethasone treatment groups, the IOP in the studied eye changed from 19.7 mmHg to 10.2 mmHg.

**Fig 2:**
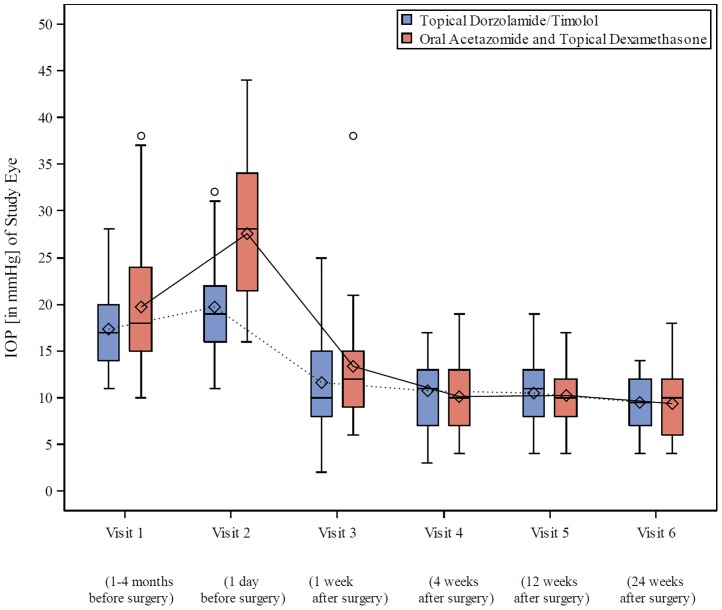
IOP (in mmHg) of the studied eye across visits–PP population (n = 62).

Non-inferiority of topical dorzolamide/timolol compared to oral acetazolamide and topical dexamethasone were demonstrated in terms of decreased IOP as measured three months after trabeculectomy. Patients in the dorzolamide/timolol group did not exhibit higher IOP than that in patients in the acetazolamide/dexamethasone group. The IOP has been measured six months after trabeculectomy analogously with no relevant differences in IOP reduction between treatments (Table 4 and Fig 2).

**Table 4:** Differences in IOP increase and decrease between the treatment groups.

	Dorzolamide / timolol	Acetazolamide / dexamethasone	Adjusted mean difference
IOP Visit 2 (1 day before surgery)	19.7 ±5.87 mmHg	27.6 ±8.02 mmHg	-6.31 mmHg; p = 0.0007 (treatment effect, PP population); 95% CI [-9.81; -2.81]
adjusted mean IOP reduction (3 months after surgery)	-8.12 mmHg	-8.30 mmHg	0.18 mmHg; p = 0.8662 (test for treatment difference); p = 0.0003 (non-inferiority test, PP population); 95% CI [-1.91; 2.26]
adjusted mean IOP reduction (6 months after surgery)	-9.13 mmHg	-9.06 mmHg	-0.07 mmHg; p = 0.9401, PP population; 95% CI [-1.85; 1.71]

The only difference between the groups was detected at visit 2 (one day before surgery). The increase in IOP (study eye) was even lower in patients in the dorzolamide/timolol group (Table 4 and Fig 2). The average IOP in the acetazolamide/dexamethasone group was remarkably higher (p = 0.0001) than that in the dorzolamide/timolol group. A subgroup analysis including an IOP baseline category (IOP < 21 mmHg vs. IOP > = 21 mmHg) one day preoperatively as a covariate in the ANCOVA model revealed no additional effect.

The total dose of oral acetazolamide per patient taken preoperatively in this group ranged from 6,000 to 26,150 mg. The number of adverse events that were probably related to the studied medication was not correlated with the amount of medication used in these patients. All adverse events except one (atrial fibrillation) were consistent with the known side-effect profile of this substance.

In addition, the ocular hypotension rate and best-corrected visual acuity (BCVA) score at baseline were analysed (Table 5). Ocular hypotension occurred in both treatment groups without any relevant differences between the groups. BCVA score at baseline and the corresponding changes to baseline (except V3 and V4) were slightly lower in the dorzolamide/timolol group than in the acetazolamide/dexamethasone group (p = 0.1914). Except for visit 5, there were no clear differences between the treatment groups according to exploratory Wilcoxon-Mann-Whitney tests. 5-FU injections to prevent cicatrisation were administered subconjunctivally when necessary. The number of 5-FU injections was similar in both treatment groups (Table 5). In some cases, it was also necessary to perform a suture lysis or a needling of the filtering bleb. The number of suture lyses was also similar between the treatment groups and the difference in needling frequency is not statistically relevant. Grading of conjunctival redness was performed using the ORA redness scale. The results were similar between the treatment groups (Table 6). The redness of the conjunctiva improved in both groups from visit 1 to visit 2. Differences in the ORA redness score between treatments per visit were compared using the Wilcoxon Mann Whitney test; no remarkable differences were observed (visit 1: p = 0.9510, visit 2: p = 0.5224 (ITT population)).

**Table 5:** Postoperative complications and treatments.

	dorzolamide/timolol group	acetazolamide/dexamethasone group
ocular hypotension*	V1+V2: No patient; V3-V6: 8 patients	
BCVA	-0.96 (±7.07); Changes to baseline at V5: Differences between treatment groups (p = 0.0372)	-4.42 (±4.33)
Number of 5-FU injections	5.26 (±3.13; range 0 to 9 or more)	5.14 (±2.61; range 0 to 9 or more)
Number of suture lyses of filtering bleb	1.22 (±1.22; range 0 to 3–4)	1.39 (±1.37; range 0 to 3–4)
Number of needlings of the bleb	2 patients	

* defined as an IOP of 0–5 mmHg

**Table 6:** Grading of conjunctiva—ITT population (N = 62).

Variable	dorzolamide/timolol (N = 30)	acetazolamide/dexamethasone (N = 32)	Total (N = 62)
ORA Score Study Eye (Visit 1)			
None	0 (0.00%)	1 (3.13%)	1 (1.61%)
Mild	12 (40.00%)	13 (40.63%)	25 (40.32%)
Moderate	15 (50.00%)	13 (40.63%)	28 (45.16%)
Severe	3 (10.00%)	4 (12.50%)	7 (11.29%)
Very Severe	0 (0.00%)	1 (3.13%)	1 (1.61%)
ORA Score Study Eye (Visit 2)			
None	7 (25.00%)	9 (33.33%)	16 (29.09%)
Mild	16 (59.26%)	15 (55.56%)	31 (57.41%)
Moderate	4 (14.29%)	2 (7.41%)	6 (10.91%)
Severe	0 (0.00%)	1 (3.70%)	1 (1.82%)
Missing*	3	5	8

* missing scores were mostly due to early study termination (before surgery took place); grading was not performed for one patient in the acetazolamide/dexamethasone group

**Fig 3:**
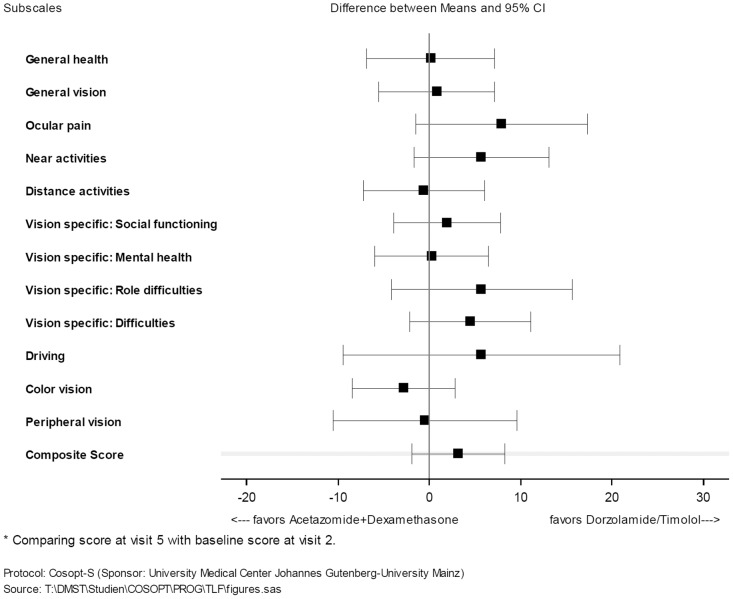
Change of NEI VFQ 25 at visit 5 from baseline (visit 2)–ITT population (n = 62).

The NEI VFQ 25 was assessed at visits 1, 2 and 5, and measures 12 aspects of vision-related quality-of-life items on a scale from 0 (poor) to 100 (excellent). A composite score was also calculated. Changes (visit 5 –visit 2) in the composite score and in the subscales were analysed using a linear model with treatment as the fixed effect and score at visit 2 as the covariate. No relevant differences were reported for the composite score or for any other score, but most of the scores favoured the treatment with topical dorzolamide/timolol (Fig 3). For some scores, a statistically significant difference was observed between the treatment groups at visit 1 (general vision p = 0.0407, near activity p = 0.0368, distance activities p = 0.0046, vision-specific mental health p = 0.0094). On average, patients in the dorzolamide/timolol group reported worse scores for general vision, near activity, distance activities and vision-specific mental health. At visit 2 (used for the ANCOVA model), a difference was detected between the treatment groups for the near activity subscore (p = 0.0419).

Epithelial defects occurred in the studied eye at all visits, no relevant differences occurred between the treatment groups. All other epithelial defects were of lower grades.. In the dorzolamide/timolol group, slightly more patients (p = 0.0161) exhibited moderate transparency and slightly fewer patients exhibited high transparency compared to the acetazolamide/dexamethasone group. The frequency of eye disorders was slightly higher in the dorzolamide/timolol group. Relevant differences regarding patterns of AEs between treatment arms were observed regarding nervous system disorders, gastrointestinal disorders, general disorders and administration site conditions, especially fatigue, cardiac disorders and investigationsto the disadvantage of acetazolamide/dexamethasone. The frequencies of all AEs that occurred in more than 5% of patients are displayed in Table 7 below, sorted by decreasing frequency in the total group. Five AEs/SAEs led to drop-outs of 5 patients (1 in the dorzolamide/timolol group and 4 in the acetazolamide/dexamethasone arm).

**Table 7:** Adverse events—safety population (N = 59).

	Number (%) of Subjects/Events ---		
	dorzolamide/timolol----	acetazolamide/dexamethasone----	Total ----
Preferred Term	(N = 28)	(N = 31)	(N = 59)
**Subjects with any Adverse Event**	23 (82.14%)	30 (96.77%)	53 (89.83%)
**Subjects with serious AE**	3 (10.71%)	4 (12.90%)	7 (11.86%)
**Subjects with severe AE**	2 (7.14%)	12 (38.71%)	14 (23.73%)
**Subjects with related AE**	7 (25.00%)	25 (80.65%)	32 (54.24%)
**Subjects with severe related AE**	1 (3.6%)	9 (29.0%)	10 (16.95%)
**Subjects with severe study drug-related AE**	1 (3.57%	9 (29.03%)	10 (16.95%)
Visual acuity reduced	15 (53.57%)	15 (48.39%)	30 (50.85%)
Fatigue	2 (7.14%)	13 (41.94%)	15 (25.42%)
Paraesthesia	0 (0.00%)	14 (45.16%)	14 (23.73%)
Dysgeusia	0 (0.00%)	10 (32.26%)	10 (16.95%)
Conjunctival hyperaemia	1 (3.57%)	6 (19.35%)	9 (4.15%)
Dizziness	2 (7.14%)	7 (22.58%)	9 (15.25%)
Nausea	1 (3.57%)	8 (25.81%)	9 (15.25%)
Eye operation complication	4 (14.29%)	5 (16.13%)	9 (15.25%)
Corneal epithelium defect	0 (0.00%)	7 (22.58%)	7 (11.86%)
Erythema of eyelid	4 (14.29%)	2 (6.45%)	6 (10.17%)
Corneal erosion	3 (10.71%)	2 (6.45%)	5 (8.47%)
Headache	2 (7.14%)	3 (9.68%)	5 (8.47%)
Abdominal pain upper	0 (0.00%)	5 (16.13%)	5 (8.47%)
Epithelial defects grade 3/severe (V3 / V4)	3 (11.54%); 0 (0.00%)	1 (3.70%); 1 (3.70%)	4 (7.55%); 1 (1.92%)
Diarrhoea	0 (0.00%)	4 (12.90%)	4 (6.78%)
Dyspnoea	0 (0.00%)	4 (12.90%)	4 (6.78%)
Eyelid oedema	1 (3.57%)	3 (9.68%)	4 (6.78%)
Intraocular pressure increased	0 (0.00%)	4 (12.90%)	4 (6.78%)
Palpitations	0 (0.00%)	4 (12.90%)	4 (6.78%)
Corneal defect	1 (3.57%)	2 (6.45%)	3 (5.08%)
Conjunctival haemorrhage	2 (7.14%)	1 (3.23%)	3 (5.08%)
Eye irritation	2 (7.14%)	1 (3.23%)	3 (5.08%)
Hypertension	2 (7.14%)	1 (3.23%)	3 (5.08%)
Hyphaema	0 (0.00%)	3 (9.68%)	3 (5.08%)

## Discussion

The primary objective of this trial was to compare the IOP reduction 3 months after trabeculectomy in a preoperative dorzolamide/timolol treatment group with that in a preoperative standard regimen with oral acetazolamide/topical dexamethasone group in patients with primary open-angle glaucoma, pseudoexfoliation glaucoma and pigmentary glaucoma to demonstrate the non-inferiority of topical dorzolamide/timolol. The primary endpoint was reached. Patients did not exhibit considerably higher IOP 3 months after surgery in the dorzolamide/timolol group compared with patients in the acetazolamide/dexamethasone group. All patients were followed up for 6 months, and similar results were detected 6 months after trabeculectomy. As expected, preoperative treatment with dorzolamide/timolol did not unfavourably affect the postoperative outcome. To the best of our knowledge, this is the first study to investigate the postoperative IOP-lowering effect of these two treatment regimens given prior to trabeculectomy.

The most common cause of post-trabeculectomy filtration failure is postoperative scarring [1]. This seems to be favoured by preoperative treatment with topical antiglaucoma agents and possibly by treatment with such medications containing preservatives [2–4]. For this reason, many surgeons, including those in our centre, prepare their patients for planned trabeculectomy by discontinuing topical antiglaucoma drugs, controlling IOP with oral acetazolamide, and treating the eye additionally with topical steroids [12]. Due to the lack of prospective trials confirming this thesis, we studied whether preoperative preparation of the study eye with the preservative-free fixed combination of dorzolamide/timolol eye drops is equally effective for postoperative IOP reduction 3 and 6 months after trabeculectomy while avoiding postoperative scarring and the known adverse side effects of oral acetazolamide and topical dexamethasone.

Öztürker et al [16] did not find any statistically significant negative influence of the preoperatively used glaucoma medications on trabeculectomy outcome. The authors found that the use of combined ß-blockers and carbonic anhydrase inhibitors, such as dorzolamide/timolol, can positively affect surgical outcome. One clear positive effect of the preoperative treatment with dorzolamide/timolol was the reduction in IOP that was observed one day before trabeculectomy; this reduction may have a protective effect on the optic nerve during the preoperative phase [13–15]. This finding suggests that regimens including individually adjustable oral medication may be inferior to fixed-dose topical medication in terms of IOP reduction. This result can be partially explained by the individualized treatment with oral acetazolamide, which is prescribed at the lowest possible dose to prevent serious side effects, possibly due to the necessity of more frequent IOP controls and dose adjustments for treatments with acetazolamide. Treatment with topical dorzolamide/timolol consists of two effective ingredients and seems to be as effective as oral acetazolamide/dexamethasone in this setting. A weakness of this study was that the dosage of oral acetazolamide was not defined in the protocol for individual patients. It was left to the investigator to decide on any increase in the amount of tablets given to the patient, depending on IOP and the disease progression. Additionally, the evaluation of IOP without the observer being blinded to the treatment group might have confounded the results. Furthermore, we did not record diurnal IOP profiles but only recorded IOP measurements once daily (always at the same time ±1 hour); these limitations should both be improved on in a future study. Another possible reason for the higher IOP one day before trabeculectomy in the acetazolamide/dexamethasone arm is that the IOP probably increased in several patients due to the treatment with topical dexamethasone 7 days preoperatively [17]. Dexamethasone was not given to patients in the dorzolamide/timolol arm for this reason, hoping that preoperative treatment with preservative-free dorzolamide/timolol would have a similar positive effect on the conjunctiva in terms of postoperative scarring. Conjunctival redness improved in both groups from visit 1 to visit 2 without relevant differences, underlining this effect. Similar results were also shown for both groups for the following postoperative parameters: classification of the filtering bleb, corneal staining, and numbers of needed treatments with 5-FU, needlings and suture lyses to prevent or treat cicatrization of the filtering bleb.

Best-corrected visual acuity (BCVA) was similar in both groups. Minor and short term worsening in BCVA, can be explained by natural post-trabeculectomy fluctuations that are induced especially by astigmatism and epithelial defects. The high number of mild epithelial effects can be explained by the side effect profile of subconjunctival 5-fluorouracil. One SAE (atrial fibrillation) occurred, which is an unknown side effect, but was judged as having a possible relationship with acetazolamide intake. The relevant differences of AE patterns between treatment arms regarding nervous system, gastrointestinal cardiac and general disorders are consistent with the side effect profile of acetazolamide [18–21]. Quality of life was assessed using the NEI VFQ-25 questionnaire. No clear differences were observed for the composite score or any subscore, but most scores favoured treatment with dorzolamide/timolol. This is probably explained by the better tolerability of the topical treatment, less frequent administration of the study treatment, and the need for fewer visits to the general practitioner and ophthalmologist. The study was executed as a prospective, active-controlled, open-label, randomized monocentric parallel group study. Masking of the patients and study personnel was not possible because of the different mode of administration of the study treatments. In a future study, a masked observer would perform the IOP measurements. Another weakness was the monocentric design of the study. On the other hand, there were only three surgeons involved in this trial, and all three performed all trabeculectomies in the same way. Fifty-three patients finished the study according to the protocol. In conclusion, regarding the risk of postoperative bleb scarring as defined as IOP reduction, the preoperative treatment with dorzolamide/timolol was not inferior to acetazolamide/dexamethasone. Moreover, IOP was not equally regulated under the individually adjusted dosage in the acetazolamide/dexamethasone group, which led to significantly higher IOP values one day before trabeculectomy. In fact, no relevant difference was observed regarding the postoperative application of 5-FU injections, suture lyses, needlings, conjunctival redness or bleb appearance between both study groups. This supports the assumption of a similar bleb scarring tendency in both treatment groups and the non-inferiority of preoperative preparation with dorzolamide/timolol compared to that with acetazolamide/dexamethasone. An interesting question remains: what would the surgical outcome have been if the control group had received only oral acetazolamide without topical dexamethasone. An additional study will be necessary in the future to show differences between the treatment of acetazolamide combined with topical dexamethasone and acetazolamide alone. This study shows that the addition of topical steroids can significantly increase IOP. With regard to systemic side effects, the acetazolamide/dexamethasone group was, as expected, inferior to topical therapy with dorzolamide/timolol. Altogether, preoperative treatment with preservative free topical dorzolamide/timolol prior to trabeculectomy seems to be a well-tolerated alternative to systemic acetazolamide/topical dexamethasone.

## Supporting information

S1 File Clinical trial protocol. (DOC)

S2 File CONSORT checklist. (DOC)
