# Supplementary material for: Efficacy and safety of preoperative IOP reduction using a preservative-free fixed combination of dorzolamide/timolol eye drops versus oral acetazolamide and dexamethasone eye drops and assessment of the clinical outcome of trabeculectomy in glaucoma
Source: PLoS One. 2017 Feb 15;12(2):e0171636. doi: 10.1371/journal.pone.0171636 (PMC5310886; doi:10.1371/journal.pone.0171636)

# CLINICAL TRIAL PROTOCOL (German Drug Law)

**Investigation of the efficacy and safety of preoperative IOP reduction with preservative-free COSOPT-S® (dorzolamide/timolol, MSD) eye drops versus oral acetazolamide and dexamethasone eye drops and assessment of the clinical outcome of trabeculectomy in glaucoma patients**

COSOPT-S*®* Study

| EudraCT 2010-019975-30  Sponsor trial code 35131  Final  Version 2.2  Date 10 DEC 2010 | |
| --- | --- |
| Sponsor  University Medical Center  Johannes Gutenberg-University Mainz  Langenbeckstrasse 1, 55131 Mainz, Germany  represented by  Univ.-Prof. Dr. Reinhard Urban  Scientific Chairman of University Medical Center  Phone +49 6131 3933180  Fax +49 6131 3933487  e-Mail wissenschaftlicher.vorstand@medizin.uni-mainz.de | Coordinating/Principal investigator  Name Dr. Katrin Lorenz, FEBO  Address Clinical Trial Site, Department of Ophthalmology, University Medical Center, Johannes Gutenberg-University Mainz, Langenbeckstr. 1, 55131 Mainz, Germany  Phone +49 6131 174069  Fax +49 6131 17474069  e-Mail lorenz@augen.klinik.uni-mainz.de |
| **Trial coordination/PV**  Name Dr. Katrin Lorenz, FEBO  Address Clinical Trial Site, Department of Ophthalmology, University Medical Center, Johannes Gutenberg-University Mainz, Langenbeckstr. 1, 55131 Mainz, Germany  Phone +49 6131 174069  Fax +49 6131 17474069  e-Mail lorenz@augen.klinik.uni-mainz.de | Biometrician/Statistician  Name Christian Ruckes  Address IZKS Mainz, Johannes Gutenberg-University Mainz, Langenbeckstr. 2, 55131 Mainz, Germany  Phone +49 6131 179919  Fax +49 6131 179914  e-Mail ruckes@izks-mainz.de |

This protocol is confidential information and is intended solely for the guidance of the clinical trial. This protocol may not be disclosed to third parties not associated with the clinical trial or used for any other purpose without the prior written consent of the sponsor.

List of abbreviations

| AE | Adverse event |
| --- | --- |
| AMG | German drug law (Arzneimittelgesetz) |
| BCVA | Best corrected visual acuity |
| CRF | Case report form |
| CTCAE | Common terminology criteria for adverse events |
| CV | Curriculum vitae |
| DSMB | Data safety monitoring board |
| EC/IEC | Ethics committee/Independent ethics committee |
| e-CRF | Electronic case report form |
| FSI | First subject in |
| GCP | Good clinical practice |
| GCP-V | GCP regulation |
| ICH | International conference on harmonization of technical requirements for registration of pharmaceuticals for human use |
| IMP | Investigational medicinal product |
| IRB | Institutional review board |
| ISRCTN | International standard randomised controlled trial number |
| INN | International nonproprietary name |
| IOP | Intraocular Pressure |
| ISF | Investigator site file |
| ITT | Intention to treat |
| KKS | Coordination Center for Clinical Trials (Koordinierungszentrum für Klinische Studien) |
| LKP | Clinical Trial Director according to AMG (Leiter der Klinischen Prüfung) |
| LSI | Last subject in |
| LSO | Last subject out |
| MedDRA | Medical dictionary for regulatory activities terminology |
| OCT | Optical Coherence Tomography |
| SAE | Serious adverse event |
| SAP | Statistical analysis plan |
| SDV | Source data verification |
| SUSAR | Suspected unexpected serious adverse reaction |
| TMF | Trial master file |

Synopsis

| Title | Investigation of the efficacy and safety of preoperative IOP reduction with preservative-free COSOPT-S® (dorzolamide/timolol, MSD) eye drops versus oral acetazolamide and dexamethasone eye drops and assessment of the clinical outcome of trabeculectomy in glaucoma patients |
| --- | --- |
| Short title | COSOPT-S®  Study |
| EudraCT | 2010-019975-30 |
| Sponsor trial code | 35131 |
| Indication | Glaucoma requiring trabeculectomy |
| Phase | IV |
| Treatments | Test product: COSOPT-S® (dorzolamide/timolol, MSD) eye drops b.i.d.  Reference therapy: oral acetazolamide 250 mg - 1500mg (DIAMOX®) per day (two or three doses), Preservative-free dexamethasone 1mg/ml eye drops five times per day |
| Primary objective | - To demonstrate that COSOPT-S® b.i.d is not inferior to the standard regimen in terms of IOP reduction three months after trabeculectomy. |
| Secondary objectives | - To show that the tolerability and safety of COSOPT-S® b.i.d. is descriptively superior to that of reference therapy - Comparison of the regimen COSOPT-S® b.i.d. with the reference regimen in terms of postoperative complications and the filtration bleb status three months after trabeculectomy - Exploration of patients’ satisfaction |
| Trial design | A prospective, active-controlled, open-label, randomized monocentric parallel group study |
| Trial population | Main inclusion criteria:  Subjects meeting all of the following criteria will be considered for enrolment to the trial:   - Male or female patients aged 18 years or older - Caucasian - A clinical diagnosis of open angle glaucoma, pseudoexfoliation or pigment dispersion glaucoma, or ocular hypertension in one or both eyes - Planned trabeculectomy - Previous treatment with antiglaucoma agents containing preservatives for at least one month - Best corrected visual acuity of 20/800 or better in the study eye   Main exclusion criteria:  Subjects presenting 1or more of the following criteria will not be enrolled in the trial:   - Secondary glaucoma except pseudoexfoliation glaucoma and pigmentary glaucoma - Current ocular infection, i.e. conjunctivitis or keratitis - Any abnormality preventing reliable applanation tonometry - Intraocular surgery or laser treatment within the past three months - History of surgery involving the conjunctiva - History of cataract surgery with sklerocorneal approach - Subject is allergic to sulfonamides - Reactive airway disease, including bronchial asthma or a history of bronchial asthma, or severe chronic obstructive pulmonary disease - Sinus bradycardia, second or third degree atrioventricular block, overt cardiac failure, cardiogenic shock - Severe renal dysfunction (CrCl < 30 ml/min) or hyperchloraemic acidosis - Depressed blood levels of sodium and / or potassium - Marked kidney and liver disease or dysfunction, gout, suprarenal gland failure, hypercalciuria or nephrocalcinosis - History of hypersensitivity to the investigational medicinal products or to any drug with similar chemical structure or to any excipient present in the pharmaceutical form of the investigational medicinal products |
| Trial duration and dates | First subject in: July 2010  Last subject out: 31 August 2011  Duration of the trial 14 months |
| Number of subjects | It is planned to enrol 60 subjects |
| Number of sites | Monocentric study |
| Primary endpoint | Mean IOP reduction (∆IOP) three months after trabeculectomy in comparison to the mean preoperative IOP |
| Secondary endpoints | - Safety and tolerability of both treatment arms: Descriptive analysis of adverse events and questionnaires. - Number of necessary 5FU injections and suture lyses at Visit 5 - Required number of needlings and reoperations at Visit 5 - Change in IOP between Visit 1 and Visit 2 - Comparison between both groups of ocular hypotension rate (IOP 0-5 mm Hg, shallow anterior chamber, Descemet’s folds, choroid swelling, macular and choroidal folds) and filtration bleb classification in both groups at every postoperative visit. - Change in quality of life for the two regimens based on the NEI VFQ 25 quality-of-life questionnaire between Visit 1, Visit 2 and Visit 5 in both groups - Patients’ satisfaction (visual analogue scale) with pharmacologic intervention for IOP lowering - Change in status of conjunctiva |
| Statistical analysis | Change in IOP between baseline and three months post-OP was defined as the primary efficacy variable. A non-inferiority test on a one-sided level of significance α=0.025 will be carried out to show that treatment with Cosopt-S® b.i.d. (7:00 am, 7:00 pm; +/- 1 h) is not inferior to the reference preoperative treatment. Δ = 4 mm Hg was specified as the non-inferiority limit. The primary analysis will be conducted on the per protocol population. |

Trial schedule

| Visit  Action | Visit 1 Screening & Randomisation | Visit 2 | Surgery | Visit 3 | Visit 4 | Visit 5  (Primary  endpoint) | Visit 6 (optional  follow-up) |
| --- | --- | --- | --- | --- | --- | --- | --- |
| Trial day | -16 weeks to -28 days | Day -1 | Day 0 | Day 7±3 | Day 28±7 | Week 12±7days | Week 24±14 days |
| Demographics (sex, age) | **X** |  |  |  |  |  |  |
| Patient information and informed consent | **X** |  |  |  |  |  |  |
| Previous and concomitant diseases | **X** |  |  |  |  |  |  |
| Previous and concomitant treatments | **X** |  |  |  |  |  |  |
| Inclusion/exclusion criteria | **X** |  |  |  |  |  |  |
| Pregnancy Test (if applicable) | **X** |  |  |  |  |  |  |
| Laboratory tests (if applicable) | **X** |  |  |  |  |  |  |
| Trabeculectomy |  |  | **X** |  |  |  |  |
| Best-corrected visual acuity (ETDRS charts) | **X** | **X** |  | **X** | **X** | **X** | **X** |
| Non-contact Pachymetry |  | **X** |  | **X** |  | **X** |  |
| IOP | **X** | **X** |  | **X** | **X** | **X** | **X** |
| Optical Coherence Tomography |  | **X** |  | **X** | **X** | **X** | **X** |
| Axial length (IOL Master) |  | **X** |  |  | **X** |  |  |
| Anterior chamber depth (AC or IOL Master) |  | **X** |  | **X** |  |  |  |
| Photography (conjunctiva) | **X** | **X** |  |  |  | **X** | **X** |
| Slit lamp examination | **X** | **X** |  | **X** | **X** | **X** | **X** |
| Randomisation | **X** |  |  |  |  |  |  |
| Filtration bleb classification (Grehn) |  |  |  | **X** | **X** | **X** | **X** |
| Grading of conjunctiva (ORA redness scale) | **X** | **X** |  |  |  |  |  |
| Questionnaires  (Patients’ satisfaction, NEI VFQ-25) | **X** | **X** |  |  |  | **X** |  |
| Previous 5-FU injections and suture lyses |  |  |  |  |  | **X** |  |
| Previous needlings |  |  |  |  |  | **X** |  |
| Changes in medical health or concomitant medication |  | **X** |  | **X** | **X** | **X** | **X** |
| Adverse events |  | **X** |  | **X** | **X** | **X** | **X** |
| End of trial (final visit) |  |  |  |  |  |  | **X** |

Trial Administrative Structure

| Sponsor (according to §4 AMG) | Name University Medical Center  Johannes Gutenberg-University Mainz  Address Obere Zahlbacherstr. 63, 55131 Mainz, Germany  Phone +49 6131 3933180  Fax +49 6131 3933487  e-Mail [wissenschaftlicher.vorstand@medizin.uni-mainz.de](mailto:wissenschaftlicher.vorstand@medizin.uni-mainz.de) |
| --- | --- |
| Sponsor’s legal representative | Name Univ.-Prof. Dr. Reinhard Urban  Scientific Chairman of University Medical Center  Address Obere Zahlbacherstr. 63, 55131 Mainz, Germany  Phone +49 6131 3933180  Fax +49 6131 3933487  e-Mail [wissenschaftlicher.vorstand@medizin.uni-mainz.de](mailto:wissenschaftlicher.vorstand@medizin.uni-mainz.de) |
| Coordinating / Principal investigator (according to §4 AMG) | Name Dr. Katrin Lorenz, FEBO  Address Department of Ophthalmology,  Johannes Gutenberg-University Mainz,  Langenbeckstr. 1, D-55131 Mainz  Phone +49 6131 174069  Fax +49 6131 175509  e-Mail lorenz@augen.klinik.uni-mainz.de |
| Trial coordinator | Name Dr. Katrin Lorenz, FEBO  Address Department of Ophthalmology,  Johannes Gutenberg-University Mainz,  Langenbeckstr. 1, D-55131 Mainz  Phone +49 6131 174069  Fax +49 6131 175509  e-Mail lorenz@augen.klinik.uni-mainz.de |
| Safety officer (SAE Management) Biometrician / Statistician  Data manager  Clinical monitor | Name IZKS Mainz  Address Johannes Gutenberg-University Mainz, Langenbeckstr. 1, 55131 Mainz, Germany  Phone +49 6131 179913  Fax +49 6131 179914 |
| Funding / grant | Name Dr. Marcus Rupp, MSD SHARP & DOHME GMBH  Address Lindenplatz 1, 85540 Haar, Germany  Phone +49 89 45 61-1380  Fax +49 89 45 61-28-1606  e-Mail markus_rupp@msd.de |

TABLE OF CONTENTS

[1 Introduction 10](#__RefHeading___Toc197240606)

[1.1 Scientific background 10](#__RefHeading___Toc197240607)

[1.2 Trial rationale 10](#__RefHeading___Toc197240608)

[1.3 Treatments and rationale for dose selection 11](#__RefHeading___Toc197240609)

[1.4 Summarized risk-benefit assessment 11](#__RefHeading___Toc197240610)

[2 Trial Objectives 12](#__RefHeading___Toc197240611)

[2.1 Primary objective 12](#__RefHeading___Toc197240612)

[2.2 Secondary objectives 12](#__RefHeading___Toc197240613)

[3 Trial Design 13](#__RefHeading___Toc197240614)

[3.1 Trial duration and schedule 13](#__RefHeading___Toc197240615)

[3.2 Number of subjects 13](#__RefHeading___Toc197240616)

[3.3 Primary endpoint 13](#__RefHeading___Toc197240617)

[3.4 Secondary endpoints 13](#__RefHeading___Toc197240618)

[3.5 Measures taken to minimize/avoid bias 14](#__RefHeading___Toc197240619)

[3.5.1 Randomisation 14](#__RefHeading___Toc197240620)

[3.5.2 Blinding 14](#__RefHeading___Toc197240621)

[3.6 Selection and withdrawal of subjects 14](#__RefHeading___Toc197240622)

[3.6.1 Inclusion criteria 14](#__RefHeading___Toc197240623)

[3.6.2 Exclusion criteria 15](#__RefHeading___Toc197240624)

[3.6.3 Withdrawal criteria 15](#__RefHeading___Toc197240625)

[3.6.4 Premature closure of trial sites 16](#__RefHeading___Toc197240626)

[3.6.5 Premature closure of the clinical trial 16](#__RefHeading___Toc197240627)

[4 TRIAL Treatments 17](#__RefHeading___Toc197240628)

[4.1 Investigational treatments 17](#__RefHeading___Toc197240629)

[4.1.1 Therapeutic effects 17](#__RefHeading___Toc197240631)

[4.1.2 Known side effects 18](#__RefHeading___Toc197240632)

[4.1.3 Dosage schedule 19](#__RefHeading___Toc197240633)

[4.1.4 Overdose instructions 20](#__RefHeading___Toc197240634)9

[4.1.5 Treatment assignment 19](#__RefHeading___Toc197240635)

[4.1.6 Treatment after the end of the trial 19](#__RefHeading___Toc197240636)

[4.1.7 Packaging and labelling 19](#__RefHeading___Toc197240637)

[4.1.8 Drug storage, supplies and accountability 21](#__RefHeading___Toc197240638)

[4.1.9 Procedures for monitoring subject compliance 21](#__RefHeading___Toc197240639)

[4.2 Not permitted medication 21](#__RefHeading___Toc197240640)

[5 TRIAL schedule 22](#__RefHeading___Toc197240642)

[6 TRial METHODS 25](#__RefHeading___Toc197240643)

[6.1 Assessment of efficacy 25](#__RefHeading___Toc197240644)

[6.2 Assessment of safety 25](#__RefHeading___Toc197240645)

[6.2.1 Adverse events 25](#__RefHeading___Toc197240646)

[6.2.1.1 Definitions 25](#__RefHeading___Toc197240647)

[6.2.1.2 Assessment of AEs by investigator 27](#__RefHeading___Toc197240648)

[6.2.1.3 Period of observation and Follow-up 28](#__RefHeading___Toc197240649)

[6.2.1.4 Documentation of AEs 28](#__RefHeading___Toc197240650)

[6.2.1.5 Immediate reporting by investigator 28](#__RefHeading___Toc197240651)

[6.2.1.6 Safety evaluation by sponsor 29](#__RefHeading___Toc197240652)

[6.2.1.7 Emergency procedures 29](#__RefHeading___Toc197240653)

[6.3 Other assessments 29](#__RefHeading___Toc197240655)

[6.3.1 Prior and concomitant illnesses 29](#__RefHeading___Toc197240656)

[6.3.2 Prior and concomitant treatments 30](#__RefHeading___Toc197240657)

[7 Statistics 31](#__RefHeading___Toc197240658)

[7.1 Sample size 31](#__RefHeading___Toc197240659)

[7.2 Analysis populations 31](#__RefHeading___Toc197240660)

[7.3 Efficacy analyses 31](#__RefHeading___Toc197240661)

[7.3.1 Definition and analysis of primary endpoint 31](#__RefHeading___Toc197240662)

[7.3.2 Analysis of secondary endpoints 32](#__RefHeading___Toc197240663)

[7.3.3 Interim analyses 32](#__RefHeading___Toc197240664)

[7.4 Analysis of adverse events 32](#__RefHeading___Toc197240665)

[8 Quality Control and Quality Assurance 32](#__RefHeading___Toc197240667)

[8.1 Requirements for investigational sites and staff 33](#__RefHeading___Toc197240668)

[8.2 Direct access to source data/documents 33](#__RefHeading___Toc197240669)

[8.3 Investigator site file and archiving 33](#__RefHeading___Toc197240670)

[8.4 Monitoring 33](#__RefHeading___Toc197240671)

[8.5 Inspection by authorities and ethic committees 34](#__RefHeading___Toc197240672)

[8.6 Audits 34](#__RefHeading___Toc197240673)

[9 Data Management 35](#__RefHeading___Toc197240674)

[9.1 Responsibilities 35](#__RefHeading___Toc197240675)

[9.2 Data collection 35](#__RefHeading___Toc197240676)

[9.3 Data handling 35](#__RefHeading___Toc197240677)

[9.4 Storage and archiving of data 36](#__RefHeading___Toc197240678)

[10 EthicAL and legal aspects 36](#__RefHeading___Toc197240679)

[10.1 Good clinical practice 37](#__RefHeading___Toc197240680)

[10.2 Patient information and informed consent 37](#__RefHeading___Toc197240681)

[10.3 Confidentiality 38](#__RefHeading___Toc197240682)

[10.4 Responsibilities of investigator 38](#__RefHeading___Toc197240683)

[10.5 Approval of trial protocol and substantial amendments 38](#__RefHeading___Toc197240684)

[10.6 Continuous information to independent ethics committee/institutional review board 39](#__RefHeading___Toc197240685)

[10.7 Submission to local regulatory/competent authorities 39](#__RefHeading___Toc197240686)

[10.8 Insurance 39](#__RefHeading___Toc197240688)

[10.9 Agreements 40](#__RefHeading___Toc197240689)

[10.9.1 Financing of the trial 40](#__RefHeading___Toc197240690)

[10.9.2 Report 40](#__RefHeading___Toc197240691)

[10.9.3 Publication policy 40](#__RefHeading___Toc197240692)

[11 Signatures 41](#__RefHeading___Toc197240693)

[12 Declaration of Investigator 42](#__RefHeading___Toc197240694)

[13 References 43](#__RefHeading___Toc197240695)

[14 Appendices 44](#__RefHeading___Toc197240696)

# Introduction

## Scientific background

The most common cause of post-trabeculectomy filtration failure is postoperative scarring [1]. Long-term preoperative treatment with local antiglaucoma agents containing preservatives, usually benzalkonium chloride, leads to histologic changes of the conjunctiva, with increased numbers and activity of lymphocytes, monocytes, fibroblasts, and beaker cells. These changes may favor the development of filtration bleb scarring [2-4] and increase the rate of post-trabeculectomy filtration failure [5-7].

Given these considerations, Broadway et al. proposed discontinuing antiglaucoma drugs and treating patients preoperatively with local steroids. Many surgeons therefore prepare their patients for planned trabeculectomy by discontinuing local antiglaucoma drugs, controlling IOP with oral acetazolamide, and treating the eye with local steroids [8]. To date no study has investigated the benefits of this approach. Consequently, preoperative treatment differs significantly between centers. Results published to date are based on retrospective, not randomized or masked, investigations, which, moreover, did not use the latest generation of local antiglaucoma drugs [9].

To pave the way for elucidating this question, we conducted a retrospective study [10] with 26 glaucoma patients who were to undergo trabeculectomy. Thirteen patients were prepared four weeks before trabeculectomy by discontinuing use of local antiglaucoma drugs in the affected eye and controlling the IOP with oral acetazolamide. One week before the procedure the eye was treated with local steroids. The other group of patients continued to receive antiglaucoma drugs up to the time of the procedure.

Trabeculectomy was conducted in all patients with intraoperative application of mitomycin C. In addition, in cases where there were signs of scarring in the postoperative observation period 5-fluorouracil was injected subconjunctivally.

IOP reduction after 12 months was identical in the two groups. The use of antiproliferative substances may have influenced this result.

We conclude from this study that discontinuation of local antiglaucoma drugs and patient preparation does not reduce the success rate of trabeculectomy. The disadvantages of current preoperative preparation and pretreatment of patients may therefore outweigh the benefits for the procedure. Firstly, the use of oral acetazolamide and local steroids often raises the IOP, which can damage the glaucomatous optic nerve [11–13]. Secondly, intolerance reactions to oral acetazolamide given over a period of four weeks are common and may be accompanied by dizziness, reduced physical performance, gastric pain, nausea, diarrhea, taste disturbances, and paresthesia resulting from potassium depletion. Compliance must therefore be monitored. Often several IOP checks by the ophthalmologist, potassium level tests by the GP, and potassium replacement are necessary.

Preservative-free COSOPT-S® eye drops b.i.d now provide a possibility to reduce IOP preoperatively with the aim of avoiding postoperative excessive scarring and the known adverse effects of oral acetazolamide while achieving comparable IOP reduction and long-term outcomes.

This study will investigate comparability (non-inferiority) of medical benefits of two different IOP lowering pharmaceutical interventions prior to trabeculectomy comparing COSOPT-S® bid to oral acetazolamide plus dexamethasone.

## Trial rationale

No treatment guidelines exist for interventions for IOP-lowering prior to trabeculectomy. This study compares head to head two commonly used pharmaceutical regimes.
This trial will investigate whether the preoperative treatment has an influence on postoperative outcome (IOP and scarring), safety and tolerability of the two interventions, whether patients prefer one of both tested therapies and health economic aspects. The results of this study will contribute a scientific basis to potential future recommendations for pharmacologic IOP-lowering interventions prior to trabeculectomy.

## Treatments and rationale for dose selection

Test product:

28 days preoperatively (± 3 days): Discontinuation of all local antiglaucomatous drugs and IOP regulation with COSOPT-S® (dorzolamide/timolol, MSD) eye drops b.i.d. (7:00 am, 7:00 pm; +/- 1 h) four weeks preoperatively.

Reference therapy:

Preoperative standard management of the Department of Ophthalmology, University Medical Center Mainz:

28 days preoperatively (± 3 days): Discontinuation of all local antiglaucomatous drugs and IOP regulation with oral acetazolamide 250 mg - 1500mg per day (two or three doses); potassium monitoring and potassium replacement if required.

7 days preoperatively (±1 day): Preservative-free dexamethasone 1mg/ml eye drops five times per day

The dosing frequency is based on that of the individual marketed products.

## Summarized risk-benefit assessment

All medicinal products which are used in this study have been used in daily practice for many years. The potential risks and benefits of the test products can be assumed to be identical to those described in the Summary of Product*.* The investigator will be informed about any relevant or new finding including AEs relating to treatment with the investigational medicinal product.

# Trial Objectives

## Primary objective

The primary objective of the trial is to investigate mean IOP reduction (∆IOP) three months after trabeculectomy in comparison to the mean preoperative IOP at Visit 1.

## Secondary objectives

The secondary objective(s) of the trial are:

- Safety and tolerability of both treatment arms: Analysis of adverse events and questionnaires.
- Number of necessary 5FU injections and suture lyses at Visit 5
- Required number of needlings and reoperations at Visit 5
- Change in IOP between Visit 1 and Visit 2
- Comparison between both groups of ocular hypotension rate (IOP 0-5 mm Hg, shallow anterior chamber, Descemet’s folds, choroid swelling, macular and choroidal folds) and filtration bleb classification in both groups at every postoperative visit
- Change in quality of life for the two regimens based on the NEI VFQ 25 quality-of-life questionnaire between Visit 1, Visit 2 and Visit 5 in both groups
- Patients’ satisfaction with pharmacologic intervention for IOP lowering between Visit 1, Visit 2 and Visit 5 in both groups
- Change in the conjunctiva status between Visit 1 and Visit 2 in both groups

# Trial Design

A prospective, active-controlled, open-label, randomized monocentric parallel group study including 60 patients.

## Trial duration and schedule

The duration of this trial is expected to be 14 months. The subject recruitment proposed to start beginning of July 2010 and end in January 2011. Last patient last visit will be achieved approximately in August 2011. However, the actual overall trial duration or subject recruitment period may vary from this time period.

## Number of subjects

It is planned to enrol 60 subjects in the clinical trial, i.e. 30 subjects per treatment group (1:1 ratio)*.* Recruitment and treatment of subjects is expected to be performed in one trial center.

## Primary endpoint

The primary objective of the trial is to investigate mean IOP reduction (∆IOP) three months after trabeculectomy (Visit 5) in comparison to the mean preoperative IOP at Visit 1.

## Secondary endpoints

- Safety and tolerability of both treatment arms: Analysis of adverse events and questionnaires.
- Number of necessary 5FU injections and suture lyses at Visit 5
- Required number of needlings and reoperations at Visit 5
- Change in IOP between Visit 1 and Visit 2
- Comparison between both groups of ocular hypotension rate (IOP 0-5 mm Hg, shallow anterior chamber, Descemet’s folds, choroid swelling, macular and choroidal folds) and filtration bleb classification in both groups at every postoperative visit.
- Change in quality of life for the two regimens based on the NEI VFQ 25 quality-of-life questionnaire between Visit 1, Visit 2 and Visit 5 in both groups
- Patients’ satisfaction with pharmacologic intervention for IOP lowering between Visit 1, Visit 2 and Visit 5 in both groups based on a visual analogue scale
- Change in the conjunctiva status between Visit 1 and Visit 2 in both groups

## Measures taken to minimize/avoid bias

In order to minimize potential bias toward the outcome of this study regarding the safety, efficacy or comfort of the test articles, patients will be randomly assigned to receive the test product or the reference therapy in the treatment phase.

### Randomisation

Patients will be randomised to COSOPT-S b.i.d. or standard therapy. Randomisation lists will be generated by IZKS Mainz by means of a computer program. The treatment allocation will be performed using central randomisation (FAX/Online or equal). The randomisation list will be kept in safe and confidential custody at the Interdisziplinäres Zentrum Klinische Studien (IZKS), Klinikum der Johannes Gutenberg-Universität, Langenbeckstr. 2, D-55131 Mainz.

### Blinding

Not applicable.

## Selection and withdrawal of subjects

No subject will be allowed to enrol in this trial more than once. Patients will be selected according to the inclusion and exclusion criteria. An elaborate informing conversation will be followed by sufficient time for making the decision. Subjects are free to discontinue the trial at any time without giving any reason.

### Inclusion criteria

Subjects meeting all of the following criteria will be considered for admission to the trial:

- Male or female patients aged 18 years or older
- Caucasian
- A clinical diagnosis of open angle glaucoma, pseudoexfoliation or pigment dispersion glaucoma, or ocular hypertension in one or both eyes
- Planned trabeculectomy
- Previous treatment with antiglaucoma agents containing preservatives for at least one month
- Best corrected visual acuity of 20/800 or better in the study eye
- Ability of subject to understand character and individual consequences of clinical trial.
- Signed and dated informed consent of the subject must be available before start of any specific trial procedures.
- Women with childbearing potential have to practicing a medically accepted contraception during trial and a negative pregnancy test (serum or urine) should be existent before trial Reliable contraception are systematic contraceptives (oral, implant, injection) and dia-phragm or condoms with spermicide. Women that are sterile by surgery or for more than two years postmenopausal can be participate in the trial.

### Exclusion criteria

Subjects presenting with any of the following criteria will not be included in the trial:

- Secondary glaucoma except pseudoexfoliation glaucoma and pigmentary glaucoma
- Current ocular infection, i.e. conjunctivitis or keratitis
- Any abnormality preventing reliable applanation tonometry
- Intraocular surgery or laser treatment within the past three months
- History of surgery involving the conjunctiva
- History of cataract surgery with sklerocorneal approach
- Subject is allergic to sulfonamides
- Reactive airway disease, including bronchial asthma or a history of bronchial asthma, or severe chronic obstructive pulmonary disease
- Sinus bradycardia, second or third degree atrioventricular block, overt cardiac failure, cardiogenic shock
- Severe renal dysfunction (CrCl < 30 ml/min) or hyperchloraemic acidosis
- Depressed blood levels of sodium and / or potassium
- Marked kidney and liver disease or dysfunction, gout, suprarenal gland failure, hypercalciuria or nephrocalcinosis
- History of hypersensitivity to the investigational medicinal products or to any drug with similar chemical structure or to any excipient present in the pharmaceutical form of the investigational medicinal products
- Participation in other clinical trials during the present clinical trial or within the last four weeks
- Medical or psychological condition that would not permit completion of the trial or signing of informed consent
- Pregnancy and lactation

A pregnancy test will be performed in women with childbearing potential prior to randomisation. Laboratory tests including all relevant values mentioned in the exclusion criteria will be performed if no current values are available.

### Withdrawal criteria

Subjects can be withdrawn their consent at their own request without given reasons at all time during the trial. This should be without disadvantages for the subject. However the investigator should try to perform a final visit to get concluding findings of investigation.

Subjects may be withdrawn from the trial for the following reasons:

- At their own request or at request of the legal representative.
- If, in the investigator’s opinion, continuation of the trial would be detrimental to the subject’s well-being.
- For women, if it becomes known that the subject is pregnant before Visit 2.
- Therapy with oral acetazolamide will be stopped if one or more of the following symptoms occur: allergic reactions (exanthema, relevant changes in blood count), renal colics, visual impairment, prolonged loss of appetite, vomiting, permanent paresthesia and /or centralnervous dysfunction (dizziness, ataxia, amentia).

The Principal investigator decides about withdrawal of subjects from the clinical trial in case of occurrence of criteria mentioned above.

In all cases, the reason for withdrawal must be recorded in the CRF and in the subject’s medical records. In case of withdrawal of a subject at his/her own request, as far as possible the reason should be asked for and documented. The subject must be followed up and as far as possible; all examinations scheduled for the final trial day should be performed and documented.

All ongoing serious adverse events of withdrawn subjects have to be followed up until no more signs and symptoms are verifiable or the health condition of the subject has stabilized, but no longer than 30 days after subjects discontinuation from the trial.

Withdrawn subjects will not be replaced.

### Premature closure of trial sites

The study center may be closed if no patients are recruited. Violation of the study protocol is another reason for premature closure of the study center. The sponsor will decide on this matter.

### Premature closure of the clinical trial

The following reasons the whole trial may be discontinued at the discretion of the sponsor:

- New risks for subjects become known.
- Inefficacy of the trial medication becomes evident.
- Occurrence of AEs unknown to date in respect of their nature, severity, and duration or the unexpected increase in the incidence of known AEs.
- Medical or ethical reasons affecting disadvantageous the continued performance of the trial.
- Difficulties in the recruitment of subjects.
- Cancellation of drug development (if applicable)

The ethic committees (EC) and the competent authorities must then be informed. Should the trial be closed prematurely, all trial material (completed, partially completed, blank CRF, randomisation envelopes, investigational medicinal products, etc.) must be returned to the sponsor or fetched by the monitor.

# TRIAL Treatments

## Investigational treatments

**Test product:**

28 days preoperatively (± 3 days): Discontinuation of all local antiglaucomatous drugs and IOP regulation with COSOPT-S® (dorzolamide 20mg/ml and timolol 5mg/ml, MSD) eye drops b.i.d. (7:00 am, 7:00 pm; +/- 1 h)

**Reference therapy:**

Preoperative standard management of the Department of Ophthalmology, University Medical Center Mainz:

28 days preoperatively (± 3 days): Discontinuation of all local antiglaucomatous drugs and IOP regulation with oral acetazolamide 250 mg - 1500mg per day (two or three doses, dosis depending on IOP); potassium monitoring and potassium replacement if required.

7 days preoperatively (±1 day): Preservative-free dexamethasone 1mg/ml eye drops five times per day

All patients will be assigned to receive either test product or reference therapy formulations as per the randomization schedule. Both treatment phases have duration of four weeks. Treatment with study medication will be discontinued at the end of the study. The investigator will give the total quantity for the treatment phase of the investigational product to the eligible subject at visit 1. At the inclusion visit, each subject will be explained in detail the mode of administration of the test product and reference therapy, as well as a follow-up notebook in which the subject should report any deviations from the stipulated frequency of application, any problems of local tolerability, any adverse reactions, and any comments related to the study that he/she may wish to add.

If the subject is unable to read, the legally acceptable representative, according to the subject’s opinion, should fill in the follow-up notebook. At the last visit, all the investigational medicinal products (full, half-used or empty) must be returned to the investigator. If the products are not returned, the investigator will question the subject to determine the reason for this.

At each visit, any change in the frequency of administration of the test product will be recorded in the Case Report Form. This evaluation will be based, in particular, on the data recorded in the follow-up notebook given to each subject.

### Therapeutic effects

Diamox ® and Cosopt S ® are used to control IOP before surgery to prevent further damage of the optic nerve. Dexa edo ® is useful to prepare the conjunctiva for trabeculectomy to prevent excessive scarring of the filtering bleb postoperatively. The conjunctiva of glaucoma patients is very irritated because most of the patients used antiglaucomatous eye drops containing preservatives for many years before a trabeculectomy is planned. Steroids without preservatives like Dexa edo ® calm down the conjunctiva. Many patients experience IOP elevations with steroids, therefore larger amounts of Diamox ® are needed in many patients in this period.

### Known side effects

The most frequent side effects associated with **dorzolamide hydrochloride** were ocular burning, stinging, or discomfort immediately following ocular administration. This occurred in about one-third of the patients taking dorzolamide hydrochloride. Approximately one-quarter of patients noted a bitter taste following administration of dorzolamide hydrochloride. Superficial punctate keratitis occurred in 10-15% of patients and side effects including an ocular allergic reaction in approximately 10%. Blurred vision, eye redness, tearing, dryness, and photophobia were side effects that occurred in 1-5% of patients taking dorzolamide hydrochloride. Other ocular events and systemic events were reported infrequently, including headache, nausea, asthenia/fatigue; and, with dorzolamide hydrochloride skin rashes, urolithiasis, and iridocyclitis will occur rarely.

Dorzolamide is a carbonic anhydrase inhibitor and although administered topically, is absorbed systemically. In clinical studies, dorzolamide was not associated with acid-base disturbances. However, these disturbances have been reported with oral carbonic anhydrase inhibitors and have, in some instances, resulted in drug interactions (e.g., toxicity associated with high-dose salicylate therapy). Therefore, the potential for such drug interactions should be considered in patients receiving dorzolamide.

Dorzolamide hydrochloride is a sulfonamide and although administered topically is absorbed systemically. Therefore, the same types of adverse reactions that are attributable to [sulfonamides](http://www.rxlist.com/script/main/art.asp?articlekey=5589) may occur with [topical](http://www.rxlist.com/script/main/art.asp?articlekey=9878) administration with dorzolamide hydrochloride. Fatalities have occurred, although rarely, due to severe reactions to sulfonamides including [Stevens-Johnson syndrome](http://www.rxlist.com/script/main/art.asp?articlekey=14086), toxic [epidermal](http://www.rxlist.com/script/main/art.asp?articlekey=32397) necrolysis, fulminant [hepatic](http://www.rxlist.com/script/main/art.asp?articlekey=3704) [necrosis](http://www.rxlist.com/script/main/art.asp?articlekey=4514), [agranulocytosis](http://www.rxlist.com/script/main/art.asp?articlekey=8816), [aplastic anemia](http://www.rxlist.com/script/main/art.asp?articlekey=2308), and other [blood](http://www.rxlist.com/script/main/art.asp?articlekey=2483) dyscrasias. Sensitization may [recur](http://www.rxlist.com/script/main/art.asp?articlekey=5255) when a sulfonamide is readministered irrespective of the route of administration. If signs of serious reactions or hypersensitivity occur, discontinue the use of this preparation.

The kidney excretes Dorzolamide hydrochloride and its metabolite predominantly. In clinical studies, local ocular adverse effects, primarily conjunctivitis and lid reactions, were reported with chronic administration of dorzolamide hydrochloride. Many of these reactions had the clinical appearance and course of an allergic-type reaction that resolved upon discontinuation of drug therapy. If such reactions are observed, dorzolamide hydrochloride should be discontinued and the patient evaluated before considering restarting the drug.

Dorzolamide hydrochloride has not been studied in patients with hepatic impairment and should therefore be used with caution in such patients. There is a potential for an additive effect on the known systemic effects of carbonic anhydrase inhibition in patients receiving an oral carbonic anhydrase inhibitor and dorzolamide hydrochloride. The concomitant administration of dorzolamide hydrochloride and oral carbonic anhydrase inhibitors is not recommended.

There have been reports of bacterial keratitis associated with the use of multiple-dose containers of topical ophthalmic products. Patients who, in most cases, had a concurrent corneal disease or a disruption of the ocular epithelial surface had inadvertently contaminated these containers. Choroidal detachment has been reported with administration of aqueous suppressant therapy (e.g., dorzolamide) after filtration procedures.

**Acetazolamide** is a sulphonamide like dorzolamide. Systemic side effects correspond to dorzolamide.

**Timolol:** Minor side effects of timolol include abdominal cramps, [diarrhea](http://www.medicinenet.com/script/main/art.asp?articlekey=24711), [constipation](http://www.medicinenet.com/script/main/art.asp?articlekey=331), fatigue, [insomnia](http://www.medicinenet.com/script/main/art.asp?articlekey=47466), [nausea](http://www.medicinenet.com/script/main/art.asp?articlekey=24732), and [vomiting](http://www.medicinenet.com/script/main/art.asp?articlekey=101642). Major side effects include [depression](http://www.medicinenet.com/script/main/art.asp?articlekey=342), vivid dreams or [memory loss](http://www.medicinenet.com/script/main/art.asp?articlekey=63547), [fever](http://www.medicinenet.com/script/main/art.asp?articlekey=361), [impotence](http://www.medicinenet.com/script/main/art.asp?articlekey=24721), lightheadedness, slow heart rate or [low blood pressure](http://www.medicinenet.com/script/main/art.asp?articlekey=1950), numbness, tingling, cramps, blueness of the hands or feet, [sore throat](http://www.medicinenet.com/script/main/art.asp?articlekey=480), and [shortness of breath](http://www.medicinenet.com/script/main/art.asp?articlekey=34434) or wheezing.

Side effects of **dexamethasone** depend on the dose, the duration and the frequency of administration. Short courses of dexamethasone usually are well tolerated with few and mild side effects. Long term, high dose dexamethasone usually will produce predictable and potentially serious side effects. Whenever possible, the lowest effective dose of dexamethasone should be used for the shortest possible length of time to minimize side effects. Alternate day dosing also can help reduce side effects.

Side effects of dexamethasone and other corticosteroids range from mild annoyances to serious irreversible damage. Side effects include fluid retention, [*weight gain*](http://www.medicinenet.com/script/main/art.asp?articlekey=24750), [*high blood pressure*](http://www.medicinenet.com/script/main/art.asp?articlekey=378), loss of potassium, [*headache*](http://www.medicinenet.com/script/main/art.asp?articlekey=20628), muscle [*weakness*](http://www.medicinenet.com/script/main/art.asp?articlekey=64119), puffiness of and hair growth on the face, thinning and [*easy bruising*](http://www.medicinenet.com/script/main/art.asp?articlekey=24714) of skin, [*glaucoma*](http://www.medicinenet.com/script/main/art.asp?articlekey=373), [*cataracts*](http://www.medicinenet.com/script/main/art.asp?articlekey=314), peptic ulceration, worsening of [*diabetes*](http://www.medicinenet.com/script/main/art.asp?articlekey=343), irregular menses, growth retardation in children, convulsions, and psychic disturbances. Psychic disturbances include [*depression*](http://www.medicinenet.com/script/main/art.asp?articlekey=342), euphoria, [*insomnia*](http://www.medicinenet.com/script/main/art.asp?articlekey=47466), mood swings, personality changes, and even psychotic behaviour.

Prolonged use of dexamethasone can depress the ability of body's adrenal glands to produce corticosteroids. Abruptly stopping dexamethasone in these individuals can cause symptoms of corticosteroid insufficiency, with accompanying [*nausea*](http://www.medicinenet.com/script/main/art.asp?articlekey=24732), [*vomiting*](http://www.medicinenet.com/script/main/art.asp?articlekey=41943), and even [*shock*](http://www.medicinenet.com/script/main/art.asp?articlekey=85053). Therefore, withdrawal of dexamethasone usually is accomplished by gradually reducing the dose. Gradually tapering dexamethasone not only minimizes the symptoms of corticosteroid insufficiency, but also reduces the risk of an abrupt flare of the disease under treatment.

Dexamethasone and other corticosteroids can mask signs of infection and impair the body's natural immune response that is important in fighting infection. Patients on corticosteroids are more susceptible to infections and can develop more serious infections than individuals not receiving corticosteroids. For example, [*chickenpox*](http://www.medicinenet.com/script/main/art.asp?articlekey=319) and [*measles*](http://www.medicinenet.com/script/main/art.asp?articlekey=6242) viruses can produce serious and even fatal illnesses in patients on high doses of dexamethasone. Live virus vaccines, such as smallpox vaccine, should be avoided in patients taking high doses of dexamethasone, since even vaccine viruses may cause disease in these patients. Some infectious organisms, such as [*tuberculosis*](http://www.medicinenet.com/script/main/art.asp?articlekey=505) (TB) and [*malaria*](http://www.medicinenet.com/script/main/art.asp?articlekey=409), can remain dormant in a patient for years. Dexamethasone and other corticosteroids can reactivate dormant infections. Patients with dormant tuberculosis may require treatment of the TB while undergoing corticosteroid treatment.

By interfering with the patient's immune response, dexamethasone can impede the effectiveness of vaccinations. Dexamethasone can also interfere with the [*tuberculin (TB) skin test*](http://www.medicinenet.com/script/main/art.asp?articlekey=26083) and cause falsely negative results in patients with dormant tuberculosis infection.

Dexamethasone impairs calcium absorption and new bone formation. Patients on prolonged treatment with dexamethasone and other corticosteroids can develop [*osteoporosis*](http://www.medicinenet.com/script/main/art.asp?articlekey=434) and an increased risk of bone fractures. Supplemental calcium and vitamin D are encouraged to slow this process of bone thinning. It has been demonstrated in some groups of patients treated with steroids that the loss of bone may be prevented by treatment with biphosphonate drugs, for example, [*alendronate*](http://www.medicinenet.com/script/main/art.asp?articlekey=753) (Fosamax).

In rare individuals, destruction of large joints can occur while undergoing treatment with dexamethasone or other corticosteroids. These patients experience severe pain in the involved joints, and can require joint replacements. The reason behind such destruction is not clear.

### Dosage schedule

**Test product:**

28 days preoperatively (± 3 days): Discontinuation of all local antiglaucomatous drugs and IOP regulation with COSOPT-S® (dorzolamide 20mg/ml and timolol 5mg/ml, MSD) eye drops b.i.d. (7:00 am, 7:00 pm; +/- 1 h).

**Reference therapy:**

Preoperative standard management of the Department of Ophthalmology, University Medical Center Mainz:

28 days preoperatively (± 3 days): Discontinuation of all local antiglaucomatous drugs and IOP regulation with oral acetazolamide 250 mg - 1500mg per day (two or three doses, dosis depending on IOP); potassium monitoring and potassium replacement if required.

7 days preoperatively (±1 day): Preservative-free dexamethasone 1mg/ml eye drops five times per day.

### Overdose instructions

Application of study medication should be stopped immediately.

**Dorzolamide hydrochloride** and **acetazolamide**: Electrolyte imbalance, development of an acidotic state, and possible central nervous system effects may occur. Serum electrolyte levels (particularly potassium) and blood pH levels should be monitored.

**Timolol:** No specific data are available at present. Expected reactions include bradycardia, hypotension, bronchospasms, acute cardiac failure, vomiting, numbness and cramps.

**Dexamethasone:** Overdose reactions did not occur so far.

### Treatment assignment

The trial medication will be administered only to subjects included in this trial.

Subjects withdrawn from the trial retain their identification codes (e.g. randomisation number). New subjects will always receive a new identification code.

### Treatment after the end of the trial

After trabeculectomy, patients receive standard postoperative treatment. IOP lowering eye drops are usually not necessary.

### Packaging and labelling

The trial medication will be handled according to §5 GCP regulation. The trial medication should be labelled with “For use in clinical trial only”. An accompanying document will be given to all participants.

### Drug storage, supplies and accountability

The investigator will take inventory and acknowledge the receipt of all shipments of the trial medication. All trial medication must be kept in a locked area with access restricted to designated trial staff.

The trial medication must be dryly stored in accordance with manufacturer’s instructions at room temperature (max. 25°C).

The investigator will also keep accurate records of the quantities of trial medication dispensed, used, and returned by each subject on the drug accountability form.

The site monitor will periodically check the supplies of trial medication held by the investigator to verify the correct accountability of all trial medication used. At the end of the trial, all unused trial medication and all medication containers will be completely returned to the sponsor if no other procedure is agreed.

It will be assured that a final drug accountability report is prepared and maintained by the investigator.

### Procedures for monitoring subject compliance

Trial medication will be dispensed to the subjects by the investigator.

Subjects will be instructed to bring all trial medication to the trial site at every visit (including all empty packages and unused trial medication). Reviewing the patient’s follow-up booklet and counting the bottles will assess compliance. Details will be recorded in the CRF and on the drug accountability form in the investigator site file.

## Not permitted medication

The following concomitant treatments are not permitted during the trial:

- - Use of any systemic medication that would affect IOP with less than a 1-month stable dosing regimen before the screening visit (i.e. steroids).
  - Use of any additional local treatment that affects IOP (except topical steroids to prevent excessive scarring).

If the subject achieves prohibited treatment, exclusion of the trial will be discussed.

Concomitant topical therapy of the non-study eye will not be documented postoperatively.

# TRIAL schedule

Visit 1 (Screening visit): -16 to -4 weeks

The following data will be obtained/checked and documented:

- Patient information and informed consent
- Age
- Gender
- Medical history and concomitant medications
- In- and exclusion criteria
- Best corrected distance visual acuity (ETDRS charts)
- IOP (GAT) (schedule to return for all visits at the same time (± 1 hour) as Visit 1.
- Slitlamp examination (biomicroscopy)
- Grading of conjunctiva (ORA redness scale)
- Photography (conjunctiva)
- Non-contact pachymetry
- Symptom survey / Questionnaires
- Urine pregnancy test if the patient is a woman of childbearing potential
- Patient instruction: patients have to discontinue their current glaucoma medication(s), starting 28 days prior to surgery
- Randomisation
- Dispense of medication, explanation of application
- Dispense of follow-up booklet
- Schedule the patient to return for the next visit, according to date of planned surgery

Visit 2: Day -1

- Changes in medical health and/or concomitant medication
- Adverse events
- Last day of administration of study drugs
- Collection of study medication after last dose
- Checking compliance
- Symptom survey / Questionnaires
- Best corrected distance visual acuity (ETDRS charts)
- IOP (GAT) at the same time as Visit 1 (± 1h)
- Non-contact pachymetry
- Optical coherence tomography
- Axial length (IOL Master)
- Anterior chamber depth (AC or IOL Master)
- Slitlamp examination (biomicroscopy)
- Grading of conjunctiva (ORA redness scale)
- Photography (conjunctiva)
- Schedule the patient to return for the next visit

**Surgery: Day 0**

- Trabeculectomy

**Visit 3: Day 7 ± 3**

- Changes in medical health and/or concomitant medication
- Adverse events
- Best corrected distance visual acuity (ETDRS charts)
- IOP (GAT) at the same time as Visit 1 (± 1h)
- Non-contact pachymetry
- Slitlamp examination (biomicroscopy)
- Filtration bleb classification (Grehn)
- Optical coherence tomography
- Anterior chamber depth (AC or IOL Master)
- Schedule the patient to return for the next visit

**Visit 4: Day 28 ± 7**

- Changes in medical health and/or concomitant medication
- Adverse events
- Best corrected distance visual acuity (ETDRS charts)
- IOP (GAT) at the same time as Visit 1 (± 1h)
- Slitlamp examination (biomicroscopy)
- Filtration bleb classification (Grehn)
- Optical coherence tomography
- Axial length (IOL Master)
- Schedule the patient to return for the next visit

**Visit 5 (Final visit): Week 12 ± 7 days**

- Changes in medical health and/or concomitant medication
- Adverse events
- Symptom survey / Questionnaires
- Best corrected distance visual acuity (ETDRS charts)
- IOP (GAT) at the same time as Visit 1 (± 1h)
- Non-contact pachymetry
- Slitlamp examination (biomicroscopy)
- Filtration bleb classification (Grehn)
- Photography (conjunctiva)
- Optical coherence tomography
- Previous 5-FU injections and suture lyses
- Previous needlings
- Schedule the patient to return for the next visit

**Visit 6 (Optional follow up): Week 24 ± 14 days**

- Changes in medical health and/or concomitant medication
- Adverse events
- Best corrected distance visual acuity (ETDRS charts)
- IOP (GAT) at the same time as Visit 1 (± 1h)
- Slitlamp examination (biomicroscopy)
- Filtration bleb classification (Grehn)
- Optical coherence tomography
- End of Trial

# TRial METHODS

## Assessment of efficacy

Intraocular pressure (IOP) will be determined on each visit (1, 2, 3, 4, 5 and 6) by means of applanation tonometry at the same time point (±1h). Change in IOP between baseline and three months post-OP was defined as the primary efficacy variable. A non-inferiority test will be carried out with α = 0.025 and β = 0.20 to show that treatment with Cosopt-S® is not inferior to the standard preoperative treatment. An ANCOVA model will be implied including the IOP baseline value as a covariate. Δ = 4 mm Hg is specified as the non-inferiority limit. The significance level α is fixed at 0.025 to account for the one-sided testing situation.

- Number of necessary 5FU injections and suture lyses at Visit 5
- Required number of needlings and reoperations at Visit 5
- Change in IOP between Visit 1 and Visit 2
- Comparison between both groups of ocular hypotension rate (IOP 0-5 mm Hg, shallow anterior chamber, Descemet’s folds, choroid swelling, macular and choroidal folds) and filtration bleb classification in both groups at every postoperative visit
- Change in quality of life for the two regimens based on the NEI VFQ 25 quality-of-life questionnaire between Visit 1, Visit 2 and Visit 5 in both groups
- Patients’ satisfaction with pharmacologic intervention for IOP lowering between Visit 1, Visit 2 and Visit 5 in both groups
- Change in the conjunctiva status between Visit 1 and Visit 2 in both groups (assessed by ORA redness scale and filtering bleb classification)

## Assessment of safety

### Adverse events

#### Definitions

Adverse Event (AE)

According to GCP, an adverse event (AE) is defined as any untoward medical occurrence in a subject treated with a pharmaceutical product and which does not necessarily have a causal relationship with this treatment. An AE can therefore be any unfavourable and unintended sign (including an abnormal laboratory finding), symptom, or disease temporally associated with the use of a medicinal investigational product, whether or not related to that product.

An AE may be:

a new symptom or medical condition

a new diagnosis

a change in laboratory parameters

an intercurrent illness or accident

worsening of a medical condition/diseases existing before the start of the clinical trial

recurrence of a disease

an increase in frequency or intensity of episodic diseases.

Surgical procedures themselves are not AEs; they are therapeutic measures for conditions that require surgery. The condition for which the surgery is required may be an AE. Planned surgical measures permitted by the clinical trial protocol and the condition(s) leading to these measures are not AEs, if the condition leading to the measure was present before inclusion in the trial. In the latter case the condition should be reported as medical history.

Change in laboratory parameters: The criteria for determining whether an abnormal test finding should be reported as an adverse event are as follows:

- Test result is associated with accompanying symptoms, and/or
- Test result requires additional diagnostic testing or medical/surgical intervention, and/or
- Test result leads to a change in trial dosing outside of protocol-stipulated dose adjustments, or discontinuation from the trial, significant additional concomitant drug treatment, or other therapy, and/or
- Test result is considered to be an adverse event by the investigator or sponsor.

Merely repeating an abnormal test, in the absence of any of the above conditions, does not constitute an adverse event. Any abnormal test result that is determined to be an error does not require reporting as an adverse event.

Change in symptoms asked in the questionnaires:

Changes in a symptom asked in the symptom survey/questionnaire will be documented as an AE if the symptom worsened at least three grades (grade 1-10). Grade 0 corresponds to no complaints.

Serious adverse event (SAE)

A serious adverse event (SAE) is one that at any dose (including overdose):

- results in death
- is life-threatening1
- requires subject hospitalization or prolongation of existing hospitalization
- results in persistent or significant disability/incapacity² or
- is a congenital anomaly/birth defect
- is a important medical event³.

1 “Life-threatening” means that the subject was at immediate risk of death at the time of the serious adverse event; it does not refer to a serious adverse event that hypothetically might have caused death if it were more severe.

2 “Persistent or significant disability or incapacity” means that there is a substantial disruption of a person’s ability to carry out normal life functions.

3 Medical and scientific judgement should be exercised in deciding whether expedited reporting is appropriate in situations where none of the outcomes listed above occurred. Important medical events that may not be immediately life-threatening or result in death or hospitalization but may jeopardize the subject or may require intervention to prevent one of the other outcomes listed in the definition above should also usually be considered serious. Examples of such events include allergic bronchospasm requiring intensive treatment in an emergency room or at home, blood dyscrasias or convulsions that do not result in subject hospitalization, or the development of drug dependency or drug abuse. A diagnosis of cancer during the course of a treatment should be considered as medically important.

Clarification of the difference in meaning between "serious" and "severe":

The terms “serious” and “severe” are not synonymous but are often used interchangeably. The term ‘severe’ is often used to describe the intensity (severity) of a specific event (as in mild, moderate, or severe myocardial infarction); the event itself, however, may be of relatively minor significance (such as severe headache). This is not the same as “serious”, which is based on subject/event outcome or action criteria usually associated with events that pose a threat to a subject’s life or functioning. Seriousness (not severity) serves as a guide for defining regulatory reporting obligations.”

#### Assessment of AEs by investigator

Intensity/Severity

The intensity of an AE will be assessed by the investigator as follows:

| Mild: | Temporary event which is tolerated well by the subject and does not interfere with normal daily activities. |
| --- | --- |
| Moderate: | Event which results in discomfort for the subject and impairs his/her normal activity. |
| Severe: | Event which results in substantial impairment of normal activities of subject. |

Causal relation to trial medication/procedures

The investigator will evaluate the causal relationship of each AE with the administration of the investigational product(s) and/or trial procedures as follows:

| Related: | A reasonable possibility that the AE may have been caused by trial participation. A related event has a close temporal relationship to trial drug administration, and an alternative cause is unlikely. E.g. the AE abates upon discontinuation of trial participation and reappears when trial participation is continued. |
| --- | --- |
| Probably: | A reasonable possibility that the event is likely to have been caused by trial participation. The AE has a temporal relationship to the trial procedure(s) and follows a known pattern of response, but a potential alternative cause may be present. |
| Possibly: | A reasonable possibility that the event may have been caused by trial participation. The AE has a temporal relationship to the trial procedure(s); but does not follow a known pattern of response, and an alternative cause seems more likely, or there is significant uncertainty about the cause of the event. |
| Unlikely: | An AE that does not follow a reasonable temporal sequence from trial participation and that is likely to have been produced by the subject’s clinical state, other modes of therapy or other known aetiology. |
| Not related: | An AE that does not follow a reasonable temporal sequence from trial participation and that is definitely caused by the subject’s clinical state, other modes of therapy or other known aetiology. |
| Not assessable: | Inadequate data for assessment, no other data may be expected. |

#### Period of observation and Follow-up

In this trial, the period of observation for collection of adverse events extends from the time the subject has signed the informed consent document up to Visit 6.

All subjects who have adverse events, whether considered associated with the use of the investigational products or not, must be monitored to determine the outcome. The clinical course of the adverse event will be followed up according to accepted standards of medical practice, even after the end of the period of observation, until a satisfactory explanation is found or the investigator considers it medically justifiable to terminate follow-up, but no longer than 30 days after the end of the trial.

Should the adverse event result in death, a full pathologist’s report should be supplied, if possible.

If the investigator detects a serious adverse event in a trial subject after the end of the period of observation, and considers the event possibly related to the prior trial, he should contact the sponsor to determine how the adverse event should be documented and reported.

#### Documentation of AEs

All AEs reported by the subject or detected by the investigator will be documented on the appropriate pages of the case report form (CRF). AEs must also be documented in the subject’s medical records.

The following approach will be taken for documentation:

- All adverse events (whether serious or non-serious) must be documented on the “Adverse Event” page of the CRF.
- If the adverse event is serious (see Section 6.2.1.1), the investigator must complete, in addition to the “Adverse Event” page, a “Serious Adverse Event” form in the CRF at the time the serious adverse event is detected.

Every attempt should be made to describe the adverse event in terms of a diagnosis. If a clear diagnosis has been made, individual signs and symptoms will not be recorded unless they represent atypical or extreme manifestations of the diagnosis, in which case they should be reported as separate events. If a clear diagnosis cannot be established, each sign and symptom must be recorded individually.

All questions on the completion and supply of adverse event report forms and any further forms issued to the investigator at a later date to clarify unresolved issues should be addressed to the sponsor.

#### Immediate reporting by investigator

SAEs must be reported immediately within 24 hours after the SAE becomes known via facsimile using the "Serious Adverse Event" form to:

| IZKS Mainz  Langenbeckstr 2  55131 Mainz  FAX 0049 6131/179916 |
| --- |

The initial SAE Report must be as complete as possible including details of subject’s identification (screening number, random number), the (serious) adverse event (medical term, diagnosis), the trial medication and an assessment of the causal relationship between the event and the trial medication made by the investigator.

The investigator should provide related additional information on the clinical course and the outcome of each SAE as soon as possible via facsimile to IZKS Mainz using the SAE form (Follow up report).

The “Serious Adverse Event” forms are provided in the Investigator Site File.

The investigator must also inform the trial monitor in all cases. The sponsor will ensure that all legal reporting requirements are met.

In addition, any pregnancy diagnosed in a female subject or in the female partner of a male subject during treatment with the investigational product must be reported to the sponsor immediately via facsimile using the SAE form.

Worsening of a sign or symptom of the condition under treatment will normally be measured by efficacy parameters. However, if the outcome fulfils the definition of “serious adverse event”, it must be reported as such.

#### Safety evaluation by sponsor

According to GCP the sponsor is responsible for the continuous safety evaluation of the investigational product(s) and the clinical trial.

The sponsor or his delegate will conduct the management of SAEs and the expedited reporting as required by German Drug Law (AMG) and GCP regulation (GCP-V). Suspected unexpected serious adverse reactions (SUSARs) and safety issues as defined by GCP-V are determined for expedited reporting: The competent authorities and the ethics committees should be notified as soon as possible but not later than 15 calendar days if the event is non-fatal and 7 calendar days if it was fatal. All investigators and the marketing authorization holder of the IMP should be informed too.

Work flow and procedures concerning SAE management will be described in a safety manual.

During a clinical trial the sponsor or his delegate will submit the annual safety report including a list of all serious adverse (drug) reactions to the ethics committee(s) and the competent authorities once a year.

#### Emergency procedures

During and following a subject’s participation in the trial, the investigator should ensure that adequate medical care is provided to a subject for any AEs including clinically significant laboratory values. The investigator should inform a subject when medical care is needed for intercurrent illness(es) of which the investigator becomes aware.

## Other assessments

### Prior and concomitant illnesses

Relevant additional illnesses present at the time of informed consent are regarded as concomitant illnesses and will be documented on the appropriate pages of the case report form (CRF).

### Prior and concomitant treatments

Relevant additional treatments administered to the subjects on entry to the trial or at any time during the trial are regarded as concomitant treatments und must be documented on the appropriate pages of the CRF. Potassium replacement might be necessary in the reference group.

# Statistics

Details of the statistical analysis of the data collected in this trial will be documented in a Statistical Analysis Plan (SAP) that will be generated by IZKS Mainz and finalized before closing the data base. The SAP is based on the protocol including all amendments. The document may modify the plans outlined in this protocol; however any major modifications of the primary endpoint definition and/or its analysis will also be reflected in a protocol amendment. Any deviation from the original statistical plan must be described and justified in the final report. The statistical analysis will be conducted by means of SAS®.

## Sample size

Estimation of sample size was based on planning a non-inferiority test for the primary efficacy criterion, i.e. the change in IOP between baseline and month 3 after the OP. A difference of 4 mm Hg between both treatment groups regarding change in IOP is considered as the limit of clinical relevance (“Delta value”).

Analysis of a pilot study including seven patients treated with Cosopt-S® and three patients under standard treatment resulted in a common standard deviation of 3.7 mm Hg for both treatment groups. Taking into account uncertainties due to the small sample size of the pilot study, it is advised to base sample size estimation on a standard deviation of 5 mm Hg.

Application of the t-test model with α = 0.025 and β = 0.20 results in 26 evaluable patients per treatment group. Considering the fact that a bit more than 10% of patients may be non-evaluable for non-inferiority, a total of 60 subjects are planned to be included in the study.

## Analysis populations

All subjects who signed informed consent are considered as enrolled subjects.

All subjects who signed informed consent and were assigned a randomisation number are considered as randomised subjects, even if they did not receive any trial treatment.

To be eligible for the per protocol population, subjects must fulfil the following criteria:

- Treatment compliance of at least 8 days.
- No concomitant treatment with medication affecting IOP (steroids, glaucoma medication)

besides the study medication.

The safety population comprises all subjects who received at least one dose of trial treatment. In analyses of the safety population subjects will be assigned to the treatment which they actually received.

The analysis populations will be defined prior to locking the database.

## Efficacy analyses

All non-inferiority hypotheses will be tested on a one-sided level of significance α=0.025. All superiority hypotheses will be tested on a two-sided level of significance α=0.05.

### Definition and analysis of primary endpoint

Change in IOP between baseline and three months post-OP was defined as the primary efficacy variable. A non-inferiority test will be carried out with to show that treatment with Cosopt-S® is not inferior to the standard preoperative treatment. An ANCOVA model will be implied including the IOP baseline value as a covariate. Δ = 4 mm Hg is specified as the non-inferiority limit. The significance level α is fixed at 0.025 to account for the one-sided testing situation. The primary analysis will be done for the per protocol population. The analysis will be repeated for the ITT population. Additionally, the 95% confidence interval for treatment differences will be displayed to check and to visualize the non-inferiority test results.

### Analysis of secondary endpoints

Two-sided significance tests with α = 0.05 will be carried out for the following secondary and tertiary efficacy variables on an exploratory basis: Change in IOP between Visit 1 and Visit 2 will be investigated by means of an ANCOVA model including the baseline value as a covariate. The number of necessary 5FU injections and sture lyses, needlings and reoperations as well as the change in vision development will be tested between groups by means of the Wilcoxon-Mann-Whitney test. The ocular hypotension rate will be compared by a Chi-Square test. The NEI VFQ 25 quality of life questionnaire (composite score and subscales) will be analysed by a linear model with repeated measurements. The same method is employed for the patients’ satisfaction. The ORA redness scale will be compared by the Wilcoxon-Mann-Whitney test. For the change in the filtering bleb classification descriptive statistics will be presented only.

All other target criteria and demographic, anamnestic and safety data will be analysed by descriptive statistical methods, i.e. quantitative data will be described by valid N, mean, standard deviation, minimum, median and maximum, qualitative and ordinal variables will be presented by means of distribution functions. Appropriate inferential statistical methods will be employed. All analyses of secondary endpoints will be interpreted purely exploratory.

### Interim analyses

No interim analyses are planned.

## Analysis of adverse events

All summaries and listings of safety data will be performed for the safety population.

Frequencies of subjects experiencing at least one adverse event (AE) will be displayed by body system and preferred term according to MedDRA terminology. Detailed information collected for each AE will include: A description of the event, duration, whether the AE was serious, intensity, relationship to trial drug, action taken, clinical outcome. Summary tables will present the number of subjects observed with AEs and corresponding percentages. Additional subcategories will be based on event intensity and relationship to trial drug.

A subject listing of all AEs will be prepared.

# Quality Control and Quality Assurance

## Requirements for investigational sites and staff

The investigator should be able to demonstrate (e.g. based on retrospective data) a potential for recruiting the required number of suitable subjects within the agreed recruitment period.

The investigator should have sufficient time to properly conduct and complete the trial within the agreed trial period.

The investigator should have available an adequate number of qualified staff and adequate facilities for the foreseen duration of the trial to conduct the trial properly and safely.

The investigator should ensure that all persons assisting with the trial are adequately qualified, informed about the protocol, any amendments to the protocol, the trials treatments, and their trial-related duties and functions.

## Direct access to source data/documents

The investigator/institution must permit trial-related monitoring and auditing by the IZKS Mainz, as well as inspections by the appropriate competent authorities and Ethics committees, providing direct access to source data/documents (Confidentiality see 10.3).

The subjects will be informed that representatives of the sponsor, independent ethics committee (IEC) or competent authorities may inspect their medical records to verify the information collected, and that all personal information made available for inspection will be handled in strictest confidence and in accordance with local data protection laws.

## Investigator site file and archiving

The investigator will be provided with an investigator site file (ISF) at the start of the trial. The investigator will archive all trial data and relevant correspondence in the ISF. The ISF, all source data and all documents will be kept filed according to the requirements of the ICH-GCP guidelines after termination of the trial.

After the end of the trial the investigator site file (ISF) and all other trial related documents e.g. trial master file (TMF) and CRFs will be stored in the archive of the sponsor.

## Monitoring

Monitoring will be done by personal visits from a clinical monitor according to SOPs of the IZKS.

To initiate the trial, the monitor will visit the participating local trial site. The monitor shall ensure that the investigators and their staff understand all requirements of the protocol and their regulatory responsibilities.

The monitor will ensure that the investigator will maintain a list of subinvestigators and other appropriately qualified persons to whom he or she has delegated significant trial-related duties (personnel log).

The site will be visited by the monitor at regular intervals to ensure compliance with the trial protocol, GCP and legal aspects. The monitor will review the entries into the CRFs for completeness and correctness and verify the entries on the basis of the source documents. The presence of correct informed consents will be checked for every subject.

Details will be specified in the monitoring manual for this trial.

The investigator must allow the monitor to look at all relevant documents and must provide support at all times to the monitor.

By frequent communications (letters, telephone, fax), the monitor will ensure that the trial is conducted according to the protocol and regulatory requirements.

## Inspection by authorities and ethic committees

Competent authorities and by the sponsor authorised persons (auditor) may request access to all source documents, CRF, and other trial documentation in case of an inspection or audit. Direct access to these documents must be guaranteed by the investigator who must provide support at all times for these activities. Source data documents can be copied during inspection or audit in case the identity of the subject have been made unrecognizable.

## Audits

No audits are planned for this trial.

# Data Management

## Responsibilities

The Data management team is authorised in the case of discrepancies or correction of data errors to contact direct the responsible person at trial site. The queries will be sent by the IZKS Mainz per fax, post or e-mail. For the response the investigator has to comply with a term defined by the IZKS. The investigator has to agree the contact per e-mail or phone.

A detailed methodology for the data management in this trial will be documented in a data management plan that will be dated and maintained by IZKS Mainz*.* This plan has to be signed by the sponsor, the head of the data management team and the responsible data manager. The document may modify the plans outlined in this protocol; however any major modifications of the data handling will also be reflected in a protocol amendment.

## Data collection

This trial will be performed using an electronic case report form (eCRF) or remote data entry (RDE). The investigator and the trial site staff will receive system documentation, training and support for the use of the eCRF. In the case of new trial site staff the training can be performed by personnel of the trial site.

For support with data entry the IZKS Mainz can be contacted.

All protocol-required information collected during the trial must be entered by the investigator, or a designated representative in the eCRF. All data entry, modification or deletion will be recorded automatically in an electronic audit trail indicating the individual subject, the original value, the new value, the reason for change, who made the change and time and date of the change. All data changes will be clearly indicated. Former values can be viewed in the audit trail. All electronic data will be entered by the site (including an electronic audit trail) in compliance with applicable record retention regulations.

The system will be secured to prevent unauthorized access to the data or the system. Only people provided with a user ID and a password will be able to enter or change data. The investigator will maintain a list of individuals who are authorized to enter or correct data and their system ID.

Computer hardware and software (for accessing the data) will be maintained at or made available for the site in compliance with applicable regulations. All technical preconditions for each trial site are record in the DMP.

The system is capable to make exact copies of data in legible paper form for inspections and audits. The investigator, following review of the data in the eCRF, will confirm the validity of each subject’s data by electronic signature or by signing a paper printout of a listing of all subjects enrolled in the trial.

The architecture of the computer system will be described in the data management plan.

## Data handling

During data entry integrity checks help to minimize entry failures. This data entry checks are based on the data validation plan, signed by the coordinating/principal investigator. The data entry system allows the trial monitors to control the entry process with the help of the system-own review functions. Comments and requests can be processed by the trial site just in time.

After completion of data entry the database access will be inhibited and the database will be exported into the data transformation system as mother database.

After completion of data entry the access for data entries is blocked and checks for plausibility, consistency and completeness of the data will be performed. Based on these checks, queries will be produced. Any missing data or inconsistencies will be reported back to the respective site and clarified by the responsible investigator. If no further corrections are to be made in the database it will be declared closed and used for statistical analysis.

All data management activities will be done according to the current Standard Operating Procedures (SOPs) of IZKS Mainz*.*

## Storage and archiving of data

According to GCP, the investigator will archive all trial data (subject identification list, source data) and relevant correspondence in the Investigator Site File (ISF). The ISF, all source data and all documents itemized in section 8 of the ICH Consolidated Guideline on GCP will be archived after finalization of the trial according to the legal regulations.

Responsible for storage and archiving of the trial data (source data and CRFs) will be the principal investigator. After completion of the trial all electronic data will be handed over to the sponsor.

# EthicAL and legal aspects

## Good clinical practice

The procedures set out in this trial protocol, pertaining to the conduct, evaluation, and documentation of this trial, are designed to ensure that all persons involved in the trial abide by good clinical practice (GCP) and the ethical principles described in the Declaration of Helsinki. The trial will be carried out in keeping with local legal and regulatory requirements.

The requirements of the AMG, the GCP regulation, and the Federal Data Protection Law (BDSG) will be kept.

## Patient information and informed consent

Before being admitted to the clinical trial, the subject must consent to participate after being fully informed about the nature, scope, and possible consequences of the clinical trial.

The documents must be in a language understandable to the subject and must specify who informed the subject.

A copy of the signed informed consent document must be given to the subject. The original signed consent document will be retained by the investigator.

The investigator will not undertake any measures specifically required only for the clinical trial until valid consent has been obtained.

If the subject has a primary physician the investigator should inform the subject’s primary physician about the subject’s participation in the trial and if the subject agrees to the primary physician being informed.

After reading the informed consent document, the subject must give consent in writing. The subject's consent must be confirmed by the personally dated signature of the subject and by the personally dated signature of the person conducting the informed consent discussions.

If the subject is unable to read, oral presentation and explanation of the written informed consent form and information to be supplied to subjects must take place in the presence of an impartial witness. Consent must be confirmed orally and by the personally dated signature of the subject or by a local legally recognized alternative (e.g., the subject's thumbprint or mark). The witness and the person conducting the informed consent discussions must also sign and personally date the consent document.

If a subject is not in a position to give informed consent because of his or her physical or mental condition, the consent of a legally authorized representative* must be sought. The consent must be confirmed at the time of consent by the personally dated signature of the representative and by the personally dated signature of the person conducting the informed consent discussions. A copy of the signed consent document must be given to the representative. The original signed consent document will be retained by the investigator. Local legal requirements must be observed and informed consent must be sought from the subject as soon as possible afterwards, if feasible. This procedure must have prior agreement from the independent ethics committee.

Footnote to define “legally authorized representative”

* “Legally authorized representative” means an individual or judicial or other body authorized under applicable law to consent on behalf of a prospective subject to the subject’s participation in the procedure(s) involved in the research.

## Confidentiality

The name of the subjects and other confidential information will not be supplied to the sponsor. During the clinical trial, subjects will be identified solely by means of an individual identification code (e.g. subject number, randomisation number). Trial findings stored on a computer will be stored in accordance with local data protection laws and will be handled in strictest confidence. For protection of these data, organizational procedures are implemented to prevent distribution of data to unauthorized persons. The appropriate regulations of data legislation will be fulfilled in its entirety.

The name of the subjects and other confidential information are subject to medical professional secrecy and the regulations of the German law on data protection (Bundesdatenschutzgesetz). During the clinical trial, subjects will be identified solely by means of an individual identification code (e.g. subject number, randomisation number). Trial findings stored on a computer will be stored in accordance with local data protection law and will be handled in strictest confidence. For protection of these data, organizational procedures are implemented to prevent distribution of data to unauthorized persons. The appropriate regulations of data legislation will be fulfilled in its entirety.

The subject will declare in the written consent to release the investigator from the medical professional secrecy to allow identification of subject’s name and/or inspection of original data for monitoring purposes by health authorities and authorized persons (monitors).

The investigator will maintain a personal subject identification list (subject numbers with the corresponding subject names) to enable records to be identified.

## Responsibilities of investigator

The investigator will ensure that all persons assisting with the trial are adequately informed about the protocol, any amendments to the protocol, the trial treatments, and their trial-related duties and functions.

The investigator will maintain a list of subinvestigators and other appropriately qualified persons to whom he or she has delegated significant trial-related duties.

Any changes of the authorized trial personnel are to be communicated without delay to the IZKS Mainz*.*

## Approval of trial protocol and substantial amendments

Before the start of the trial, the trial protocol, informed consent document, and any other appropriate documents will be submitted to the independent ethics committee (IEC)/institutional review board (IRB). Approval (respectively formal approval) by the IEC should preferably mention the title of the trial, the trial code, if applicable the trial site, and the documents they reviewed. It must mention the date on which the decision was made and must be officially signed by a committee member. This documentation must also include a list of members of the IEC present on the applicable EC meeting.

If applicable, the documents will also be submitted to the competent authorities (approval by the highest competent authority, BfArM), in accordance with the respective local legal requirements.

Investigational products can only be supplied to the investigator after documentation on all ethical and legal requirements for starting the clinical trial has been received by the sponsor (“regulatory greenlight”). Before the first subject is enrolled in the trial, all ethical and legal requirements must be met.

Neither the investigator nor the sponsor will alter this trial protocol without obtaining the written agreement of the other. The IEC and, if applicable, the competent authorities must be informed of all subsequent protocol amendments and administrative changes, in accordance with the respective local legal requirements.

Amendments must be evaluated to determine whether formal approval must be sought and whether the informed consent document should also be revised.

The investigator must keep a record of all communications with the IEC and the competent authorities.

## Continuous information to independent ethics committee/institutional review board

The EC must be informed of all subsequent protocol amendments which require formal approval in accordance with the legal requirements.

The independent EC must be informed of serious or unexpected AEs according to the current legal requirements which occur during the trial and might affect the safety of subjects or the conduct of the trial if not otherwise stated in the vote.

The EC must be informed of trial process regularly if not otherwise stated in the vote. The EC must be informed of the end of the trial in accordance with legal requirements (within 90 days or within 15 days in case of premature closure of the clinical trial).

The independent EC must be informed of all subsequent protocol amendments and administrative changes, in accordance with local legal requirements.

Unless otherwise instructed by the independent EC/institutional review board (IRB), the investigator must submit to the EC:

- Information on serious or unexpected adverse events from the investigator’s site, as soon as
 possible.

- Expedited safety reports from the sponsor.

- Periodic reports on the progress of the clinical trial.

## Submission to local regulatory/competent authorities

Before the start of trial, the sponsor is responsible for submission of all documents necessary to the competent authorities for approval. The local regulatory authority responsible for the investigator will be informed of the trial.

## Insurance

According to § 40 AMG, the sponsor has to subscribe to an insurance policy covering, in its terms and provisions, its legal liability for injuries caused to participating persons and arising out of this research performed strictly in accordance with the scientific protocol as well as with applicable law and professional standards. The insurance will be taken out at Ecclesia Versischerungsdienst.

Any impairment of health which might occur in consequence of trial participation must be notified to the insurance company. The subject is responsible for notification. The insured person will be agreed to all appropriate measures serving for clarification of the cause and the extent of damage as well as the reduction of damage.

During the conduct of the trial, the subject must not undergo other clinical treatment except for cases of emergency. The subject is bound to inform the investigator immediately about any adverse events and additionally drugs taken. The terms and conditions of the insurance should be delivered to the subject.

Insurance provisions for this clinical trial are given in separate agreements.

## Agreements

### Financing of the trial

This Investigator Initiated Trial is supported financially by MSD SHARP & DOHME GMBH, Lindenplatz 1, 85540 Haar, Germany.

The general conditions of financing for this trial are given in separate agreements.

### Report

After conclusion of the trial, a report shall be written by the sponsor or his delegate, in cooperation with the coordinating investigator. The report will include a statistical analysis and an appraisal of the results from a medical viewpoint. It will be based on the items listed in this trial protocol. Evaluation of pharmacoeconomic results will be performed by MSD SHARP & DOHME GMBH, Lindenplatz 1, 85540 Haar, Germany.

### Publication policy

Any publication of the results, either in part or in total (articles in journals or newspapers, oral presentation, etc.) by the investigators will be submitted to the sponsor at least sixty (60) days prior to submission. It is planned to publish the results of the trial as an original article in an appropriate medical journal as well as presentation at congresses. The Principal Investigator Dr. Katrin Lorenz is first author of the article and will present the data at the major congresses. The first author in agreement with the co-authors will make the choice of the journal for the publication. Besides the Principal Investigator, further authors of this article have to meet the following points:

- substantial contribution to the recruitment of subjects
- substantial contribution to interpretation of the data.
- substantial contribution to drafting the article or revising it critically for important intellectual content.

# Signatures

The present trial protocol was subject to critical review and has been approved in the present version by the persons undersigned. The information contained is consistent with:

- the current risk-benefit assessment of the investigational medicinal product.
- the moral, ethical, and scientific principles governing clinical research as set out in the Declaration of Helsinki and the principles of GCP.

Sponsor

| Name Univ.-Prof. Dr. Reinhard Urban  _________________ _____________________________________ |
| --- |
| Date Signature |
|  |
| Coordinating/Principal Investigator  Name Dr. Katrin Lorenz, FEBO |
| _________________ _____________________________________ |
| Date Signature |
|  |
| Trial coordination  Name Dr. Katrin Lorenz, FEBO |
| _________________ _____________________________________ |
| Date Signature |
|  |
| Statistician  Name Dipl. Math. Christian Ruckes |
| _________________ _____________________________________ |
| Date Signature |

# Declaration of Investigator

I have read the above trial protocol and I confirm that it contains all information to accordingly conduct the clinical trial. I pledge to conduct the clinical trial according to the protocol.

I will enrol the first subject only after all ethical and regulatory requirements are fulfilled. I pledge to obtain written consent for trial participation from all subjects.

I know the requirements for accurate notification of serious adverse events and I pledge to document and notify such events as described in the protocol.

I pledge to retain all trial-related documents and source data as described. I will provide a Curriculum Vitae (CV) before trial start. I agree that the CV may be submitted to the responsible competent authorities.

I will conduct the trial in compliance with the protocol, GCP and the applicable regulatory requirements.

Investigator

Name Dr. Katrin Lorenz, FEBO

Address Department of Ophthalmology, University Medical Center, Johannes Gutenberg-University Mainz, Langenbeckstr. 1, 55131 Mainz, Germany

Phone +49 6131 174069

Fax +49 6131 17474069

e-Mail lorenz@augen.klinik.uni-mainz.de

| _________________ _____________________________________ |
| --- |
| Date Signature |

Subinvestigator

Name PD Dr. Hagen Thieme

Address Department of Ophthalmology, University Medical Center, Johannes Gutenberg-University Mainz, Langenbeckstr. 1, 55131 Mainz, Germany

Phone +49 6131 175445

Fax +49 6131 176620

e-Mail [thieme@augen.klinik.uni-mainz.de](mailto:thieme@augen.klinik.uni-mainz.de)

| _________________ _____________________________________ |
| --- |
| Date Signature |

# References

1. Skuta GL, Parrish, RK (1987) Wound healing in glaucoma filtering surgery. Surv Ophthalmol 32(3):149-170

2. Baudouin C, Pisella PJ, Fillacier K, Goldschild M, Becquet F, De Saint Jean M, Bechetoille A (1999) Ocular surface inflammatory changes induce by topical antiglaucoma drugs: human and animal studies. Ophthalmology, 106: 556-563

3. Broadway DC, Grieson I, O’Brien C, Hitchings RA (1994) Adverse effects of topical antiglaucoma medication. II. The outcome of filtration surgery. Arch Ophthalmol, 112: 1446-1454

4. Sherwood MB, Grierson I, Millar L, Hitchings RA (1989) Long-term morphologic effects of antiglaucoma drugs on the conjunctiva and Tenon’s capsule in glaucomatous patients. Ophthalmology, 96: 327-335

5. Broadway DC, Grierson I, O’Brien C, Hitchings RA (1994) Adverse effects of topic antiglaucoma medication I. Conjunctiva profile. Arch Ophthalmol, 112: 1437-1445

6. Lavin MJ, Wormald RP, Migdal CS, Hitchings RA (1990) The influence of prior therapy on the success of trabeculectomy. Arch Ophthalmol, 108: 1543-1548

7. Longstaff S Wormald RPL, Mazover A, Hitchings RA (1990) Glaucoma triple procedures. Ophthalmic Surg 21: 786 - 793

8. Broadway DC, Grierson I, Stürmer J, Hitchings RA (1996) Reversal of Topical Antiglaucoma Medication. Effects on the Conjunctiva. Arch Ophthalmol, 114: 262 – 267

9. Flach, AJ (2004) Does medical treatment influences the success of trabeculetomy. Trans AM Ophthalmolg Soc, 102: 219-224

10. Keicher A, Wahl J, Pfeiffer N (2006): Influence of local glaucoma therapy on rate of success of trabeculectomy, DOG 2006 Berlin

11. Leibowitz HM, Bartlett JD, Rich R, McQuirter H, Stewart R, Assil K ( 1996) Intraocular pressure-raising potential of 1.0% rimexolone in patients responding to corticosteroids. Arch Ophthalmol, 114(8):933-937

12. Zeimer RC, Wilensky JT, Gieser DK, Viana MAG (1990) Association between intraocular pressure peaks and progression of visual field loss. Ophalmol, 98: 64 – 69

13. Faibairn WD, Thorson JC (1971) Fluorometholone: anti-inflammatory and intraoccular pressure effects. Arch Ophthalmol 86: 138 – 141

# Appendices

**14.1 Questionnaire 1**

Sehr geehrte Patientin, sehr geehrter Patient,

die folgenden Fragen betreffen Ihr Sehvermögen und Ihre persönliche Zufriedenheit mit Ihrem Sehen. Bitte wählen Sie diejenige Antwort, die am besten zutrifft.

Nehmen Sie sich für jede Frage soviel Zeit, wie Sie benötigen. Wir möchten so gut wie möglich verstehen, wie Ihre Lebensqualität durch Ihr Sehvermögen beeinflusst wird. Die Fragen sollten daher so genau wie möglich beantwortet werden.

Falls Sie Brillen- oder Kontaktlinsenträger sind: Bitte beantworten Sie die Fragen so, als ob Sie Ihre Brille oder Ihre Kontaktlinsen tragen würden.

Anleitung:

1. Es wäre am besten, wenn Sie den Fragebogen ohne fremde Hilfe ausfüllen.
2. Beantworten Sie bitte alle Fragen.
3. Kreuzen Sie die zutreffende Antwort an.
4. Wenn Sie sich bei einer Frage nicht ganz sicher sind: Geben Sie bitte die am ehesten zutreffende Antwort. Sie können an den Rand einen Kommentar schreiben.

Vielen Dank für Ihre Teilnahme.

**I. Allgemein**

**1. Wie schätzen Sie Ihre Gesundheit im Allgemeinen ein?**

- Sehr gut
- Gut
- Befriedigend
- Ausreichend
- Schlecht

**2. Wie beurteilen Sie Ihr derzeitiges Sehen mit beiden Augen (mit Brille oder Kontaktlinsen, falls Sie diese tragen)?**

- Sehr gut
- Gut
- Befriedigend
- Ausreichend
- Schlecht
- Blind

**3. Wie häufig machen Sie sich Gedanken über Ihr Sehvermögen?**

- Gar nicht
- Selten
- Manchmal
- Sehr oft
- Immer

**4. Haben Sie Schmerzen oder andere Beschwerden, wie Brennen, Kratzen oder Jucken, in den Augen oder im Augenbereich?**

- Keine
- Geringe
- Mäßige
- Starke
- Sehr starke

**II. Probleme im Alltag**

**5. Wie schwer fällt es Ihnen, die normale Schriftgröße einer Zeitung zu lesen?**

- Überhaupt keine Schwierigkeiten
- Ein wenig Schwierigkeiten
- Deutliche Schwierigkeiten
- Sehr starke Schwierigkeiten
- Wegen meiner Sehschwäche kann ich das nicht mehr
- Ich lese (aus anderen Gründen) keine Zeitung

**6. Welche Schwierigkeiten haben Sie bei Alltagsbeschäftigungen, die ein gutes Sehen in der Nähe erfordern, wie z.B. Kochen, Nähen oder die Arbeit mit Werkzeugen?**

- Überhaupt keine Schwierigkeiten
- Ein wenig Schwierigkeiten
- Deutliche Schwierigkeiten
- Sehr starke Schwierigkeiten
- Wegen meiner Sehschwäche kann ich das nicht mehr
- Ich gehe diesen Tätigkeiten (aus anderen Gründen) nicht nach

**7. Welche Schwierigkeiten haben Sie aufgrund Ihres Sehvermögens, in einem voll gestellten (überfüllten) Regal etwas zu finden?**

- Überhaupt keine Schwierigkeiten
- Ein wenig Schwierigkeiten
- Deutliche Schwierigkeiten
- Sehr starke Schwierigkeiten
- Wegen meiner Sehschwäche kann ich das nicht mehr
- Ich suche keine Dinge in Regalen

**8. Wie schwer fällt es Ihnen, Straßenschilder oder die Namen von Geschäften zu erkennen?**

- Überhaupt keine Schwierigkeiten
- Ein wenig Schwierigkeiten
- Deutliche Schwierigkeiten
- Sehr starke Schwierigkeiten
- Wegen meiner Sehschwäche kann ich das nicht mehr
- Trifft auf mich nicht zu

**9. Welche Schwierigkeit bereitet es Ihnen aufgrund Ihres Sehens, in der Dämmerung oder in der Nacht Stufen und Treppen hinab zu steigen?**

- Überhaupt keine Schwierigkeiten
- Ein wenig Schwierigkeiten
- Deutliche Schwierigkeiten
- Sehr starke Schwierigkeiten
- Wegen meiner Sehschwäche kann ich das nicht mehr
- Trifft auf mich nicht zu

**10. Welche Schwierigkeiten haben Sie aufgrund Ihres Sehens, Dinge im Vorbeigehen zu erkennen?**

- Überhaupt keine Schwierigkeiten
- Ein wenig Schwierigkeiten
- Deutliche Schwierigkeiten
- Sehr starke Schwierigkeiten
- Wegen meiner Sehschwäche kann ich das nicht mehr
- Trifft auf mich nicht zu

**11. Welche Schwierigkeiten bereitet es Ihnen aufgrund Ihres Sehens, die Reaktion von Menschen zu beurteilen, wenn Sie diese ansprechen?**

- Überhaupt keine Schwierigkeiten
- Ein wenig Schwierigkeiten
- Deutliche Schwierigkeiten
- Sehr starke Schwierigkeiten
- Wegen meiner Sehschwäche kann ich das nicht mehr
- Trifft auf mich nicht zu

**12. Welche Schwierigkeiten bereitet es Ihnen aufgrund Ihres Sehens, Kleidung aus dem Schrank zu nehmen und mit anderen Kleidungsstücken zu kombinieren?**

- Überhaupt keine Schwierigkeiten
- Ein wenig Schwierigkeiten
- Deutliche Schwierigkeiten
- Sehr starke Schwierigkeiten
- Wegen meiner Sehschwäche kann ich das nicht mehr
- Trifft auf mich nicht zu

**13. Welche Schwierigkeiten haben Sie aufgrund Ihres Sehens bei Einladungen oder im Restaurant?**

- Überhaupt keine Schwierigkeiten
- Ein wenig Schwierigkeiten
- Deutliche Schwierigkeiten
- Sehr starke Schwierigkeiten
- Wegen meiner Sehschwäche gehe ich auf keine Einladungen / ins Restaurant
- Trifft auf mich nicht zu

**14. Welche Schwierigkeiten haben Sie aufgrund Ihres Sehens Kinofilme, Theaterstücke oder Sportveranstaltungen anzuschauen?**

- Überhaupt keine Schwierigkeiten
- Ein wenig Schwierigkeiten
- Deutliche Schwierigkeiten
- Sehr starke Schwierigkeiten
- Wegen meiner Sehschwäche kann ich das nicht mehr
- Trifft auf mich nicht zu

**15. Fahren Sie derzeit Auto?**

- Ja 🡺 gehen Sie zu Frage 15c
- Nein

**15a**. Falls Nein: Sind Sie nie Auto gefahren oder haben Sie aufgehört, Auto zu fahren?

- Nie Auto gefahren 🡺 gehen Sie zu Frage 17
- Aufgehört

**15b**. Wenn Sie aufgehört haben, Auto zu fahren: Was war der hauptsächliche Grund dafür?

- Aufgrund meiner Sehschwäche 🡺 gehen Sie zu Frage 17
- Wegen anderer Gründe 🡺 gehen Sie zu Frage 17
- Wegen meiner Sehschwäche und aus anderen Gründen 🡺 gehen Sie zu Frage 17

**15c**. Wenn Sie derzeit Auto fahren: Welche Schwierigkeiten bereitet es Ihnen, während des Tages in bekannter Umgebung Auto zu fahren?

- Überhaupt keine Schwierigkeiten
- Ein wenig Schwierigkeiten
- Deutliche Schwierigkeiten
- Sehr starke Schwierigkeiten

**16. Welche Schwierigkeiten haben Sie mit dem Autofahren in der Nacht?**

- Überhaupt keine Schwierigkeiten
- Ein wenig Schwierigkeiten
- Deutliche Schwierigkeiten
- Sehr starke Schwierigkeiten
- Wegen meiner Sehschwäche kann ich nachts nicht mehr Auto fahren
- Trifft auf mich nicht zu

**16a**. Welche Probleme bereitet es Ihnen, in schwierigen Situationen (schlechtes Wetter, Berufsverkehr, Stau) Auto zu fahren?

- Überhaupt keine Schwierigkeiten
- Ein wenig Schwierigkeiten
- Deutliche Schwierigkeiten
- Sehr starke Schwierigkeiten
- Wegen meiner Sehschwäche kann ich in diesen Situationen nicht mehr Auto fahren
- Trifft auf mich nicht zu

**III. Generelle Probleme mit dem Sehen**

**17. Schaffen Sie aufgrund Ihres Sehvermögens weniger als Sie möchten?**

- Immer
- Meistens
- Manchmal
- Selten
- Nie

**18. Können Sie, bedingt durch Ihr Sehen, nicht mehr so lange arbeiten oder Dinge erledigen?**

- Immer
- Meistens
- Manchmal
- Selten
- Nie

**19. Werden Sie durch Schmerzen oder andere Beschwerden (Brennen, Kratzen oder Jucken) in den Augen oder im Augenbereich von dem, was Sie tun wollen, abgehalten?**

- Immer
- Meistens
- Manchmal
- Selten
- Nie

**20. Wegen meiner Sehschwäche bleibe ich die meiste Zeit zuhause.**

- Trifft voll zu
- Trifft meistens zu
- Ab und zu
- Eigentlich nicht
- Überhaupt nicht

**21. Wegen meiner Sehschwäche bin ich oft deprimiert.**

- Trifft voll zu
- Trifft meistens zu
- Ab und zu
- Eigentlich nicht
- Überhaupt nicht

**22. Wegen meiner Sehschwäche habe ich viele Dinge nicht mehr unter Kontrolle.**

- Trifft voll zu
- Trifft meistens zu
- Ab und zu
- Eigentlich nicht
- Überhaupt nicht

**23. Wegen meiner Sehschwäche muss ich zu oft darauf vertrauen, was andere Menschen mir sagen.**

- Trifft voll zu
- Trifft meistens zu
- Ab und zu
- Eigentlich nicht
- Überhaupt nicht

**24. Wegen meiner Sehschwäche bin ich häufig auf die Hilfe anderer angewiesen.**

- Trifft voll zu
- Trifft meistens zu
- Ab und zu
- Eigentlich nicht
- Überhaupt nicht

**25. Ich mache mir Gedanken darüber, dass ich mich oder andere wegen meiner Sehschwäche in unangenehme Situationen bringe.**

- Trifft voll zu
- Trifft meistens zu
- Ab und zu
- Eigentlich nicht
- Überhaupt nicht

14.2 **Questionnaire 2**

**14.3 Filtration bleb classification (Grehn)**

|  | **0** = none | **1** = mild | **2** = a few Corkscrew vessels |  |
| --- | --- | --- | --- | --- |
| **Vascularisation** |  |  |  |  |
|  | **0** = no borders to the sides | **1** = demarcation nasally or temporally | **2** = demarcation to both sides | **3** = encapsulated |
| **Identifiability** |  |  |  |  |
|  | **0** = a least 3 mm | **1** = 2 mm | **2** = 1 mm | **3** = flat |
| **Thickness** |  |  |  |  |
|  | **0** = no | **1** = yes |  |  |
| **Microcysts** |  |  |  |  |
|  | **0** = highly transparent | **1** = moderate | **2** = not transparent |  |
| **Transparency** |  |  |  |  |
|  | **0** = yes | **1** = no |  |  |
| **Mobility** |  |  |  |  |
|  | **0** = yes | **2** = no |  |  |
| **Leakage** |  |  |  |  |

**14.4 ORA Redness Scale**

Redness of the conjunctiva will be graded by *ORA Redness Scale* (Grade 0-4).

0 = none (normal)

1 = mild (a flush reddish colour predominantly confined to the palbebral or bulbar conjunctiva)

2 = moderate (more prominent red colour of the palbebral or bulbar conjunctiva)

3 = severe (definite redness of palbebral or bulbar conjunctiva)

4 = very severe (severe redness of palbebral and bulbar conjunctiva)


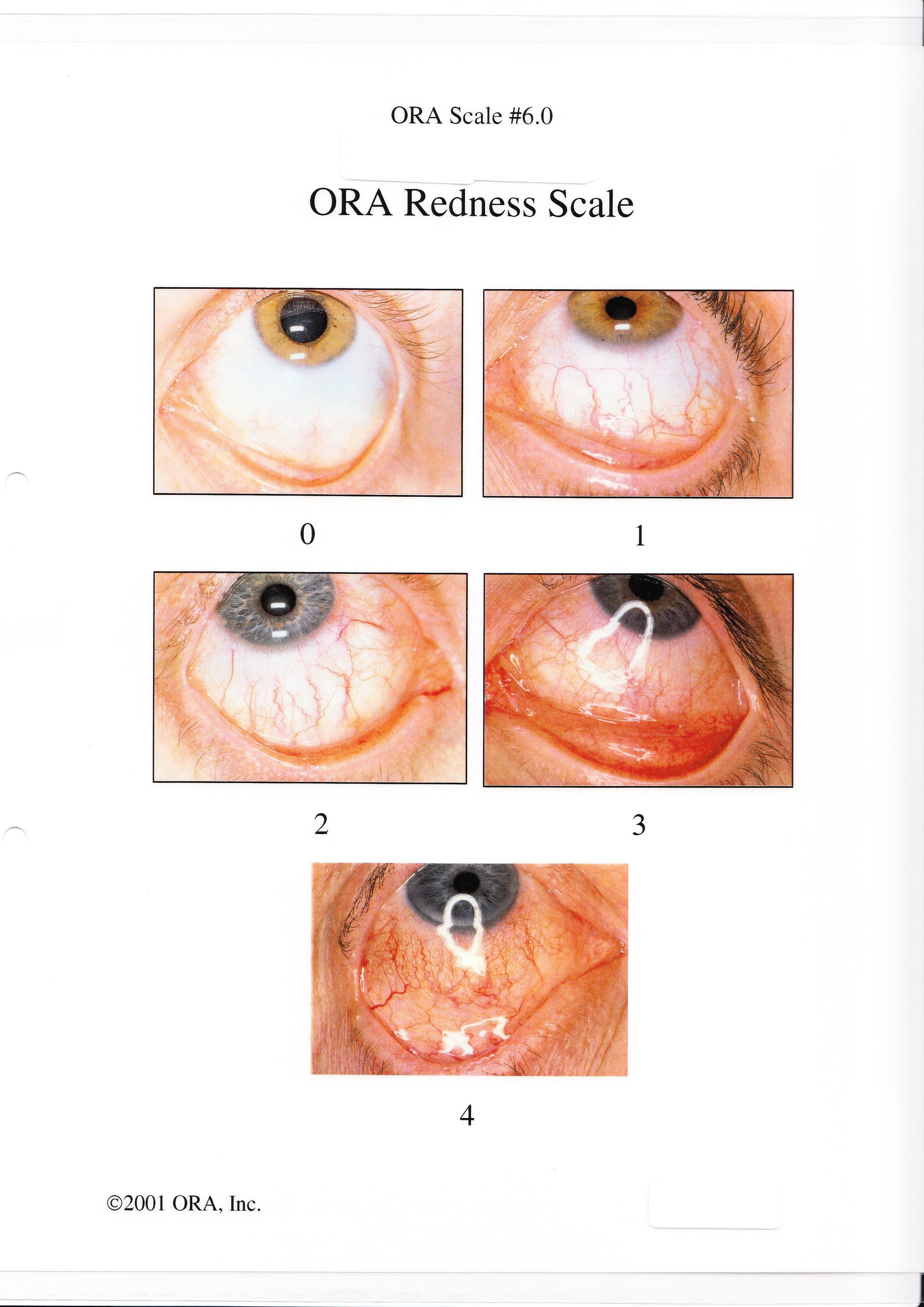

Supplement: S1 File — (DOC) [file pone.0171636.s001.doc]
